# ﻿New taxonomic and faunistic data on the funnel-weavers (Araneae, Agelenidae) of Turkiye and the Caucasus, with five new species

**DOI:** 10.3897/zookeys.1218.135249

**Published:** 2024-11-21

**Authors:** Alireza Zamani, Rahşen S. Kaya, Yuri M. Marusik

**Affiliations:** 1 Zoological Museum, Biodiversity Unit, FI-20014 University of Turku, Turku 20500, Finland University of Turku Turku Finland; 2 Department of Biology, Faculty of Arts and Science, Bursa Uludağ University, TR-16059, Bursa, Turkiye Bursa Uludağ University Bursa Turkiye; 3 Department of Zoology & Entomology, University of the Free State, Bloemfontein 9300, South Africa University of the Free State Bloemfontein South Africa; 4 Altai State University, Lenina Pr., 61, Barnaul, RF-656049, Russia Altai State University Barnaul Russia; 5 Institute for Biological Problems of the North, Portovaya Str. 18, Magadan 685000, Russia Institute for Biological Problems of the North Magadan Russia

**Keywords:** Anatolia, Armenia, Georgia, *Maimuna*, new record, new synonymy, *Persiscape*, *Tegenaria*

## Abstract

New taxonomic and faunistic data on the agelenid spiders of Turkiye and the Caucasus are provided. Five species are described as new to science: *Maimunaantalyensis***sp. nov.** (♂♀; Turkiye: Antalya), *Tegenariaballarini***sp. nov.** (♂♀; Turkiye: Antalya), *T.beyazcika***sp. nov.** (♂; Turkiye: Antalya), *T.egrisiana***sp. nov.** (♂♀; Georgia: Imereti), and *T.hoeferi***sp. nov.** (♂♀; Armenia: Kotayk). *Tegenarialazarovi* Dimitrov, 2020, **syn. nov.** is proposed as a new junior synonym of *T.averni* Brignoli, 1978. *Persiscapecaucasica* (Guseinov, Marusik & Koponen, 2005) is newly reported from Armenia, and *T.chumachenkoi* Kovblyuk & Ponomarev, 2008 is reported for the first time from Turkiye. New distribution records for *T.dalmatica* Kulczyński, 1906, *T.hamid* Brignoli, 1978, *T.longimana* Simon, 1898 and *T.percuriosa* Brignoli, 1972, and topotype material for *T.tekke* Brignoli, 1978 are reported. The record of *Eratigenafuesslini* (Pavesi, 1873) from Turkiye is found to be based on a misidentification, and is herein attributed to *T.hamid*. The presence of an embolic spine, unknown in any other species of *Tegenaria*, is documented in *T.anhela* Brignoli, 1972 for the first time. Photographs are provided for all treated species.

## ﻿Introduction

Agelenidae C.L. Koch, 1837 is a large family of spiders, encompassing 1,405 extant species across 96 genera worldwide (WSC 2024). Commonly known as “funnel-weavers,” the family has been relatively well-studied in the Palaearctic (e.g., de Blauwe 1980; Levy 1996). In the Western Palaearctic, Turkiye has the highest recorded diversity of Agelenidae, with 74 species documented (Danışman et al. 2024). Other areas within the Western Palaearctic remain largely under-studied. For example, in the Caucasus, Otto (2022) lists 36 species in ten genera of Agelenidae, yet only three species have been reported from Armenia and 18 from Georgia to date. This highlights the limited understanding of agelenid diversity in this region (Zarikian et al. 2022).

While examining spiders from Turkiye, Georgia, and Armenia, we had the opportunity to study several agelenid specimens from these countries. In this paper, we present the following findings: the descriptions of four new species of *Tegenaria* Latreille, 1804 and of one new species of *Maimuna* Lehtinen, 1967; the synonymization of *T.lazarovi* Dimitrov, 2020; the presence of an embolic spine in *T.anhela* Brignoli, 1972; and several new faunistic data for agelenids in Turkiye and Armenia.

## ﻿Materials and methods

Photographs of specimens and their copulatory organs were obtained using an Olympus Camedia E‐520 camera attached to an Olympus SZX16 stereomicroscope at the Zoological Museum of the University of Turku, Finland. Digital images of different focal planes were stacked with Helicon Focus™ 8.1.1. Illustrations of vulvae were made after digesting tissues off in a 10% KOH aqueous solution. Body measurements exclude the chelicerae and spinnerets. Leg segments were measured on the dorsal side. Measurements of legs are listed as: total (femur, patella, tibia, metatarsus, tarsus). All measurements are given in millimeters.

### ﻿Abbreviations used in the text and figures

Eyes: **ALE**—anterior lateral eye, **AME**—anterior median eye, **PLE**—posterior lateral eye, **PME**—posterior median eye.

Leg segments: **Fe**—femur, **Pa**—patella, **Ta**—tarsus, **Ti**— tibia.

Male palp: **Eb**—embolus base, **Em**—embolus, **Es**—embolic spine, **Cc**—claw-like projections of the conductor, **Cn**—conductor, **Cp**—basal process of the cymbium, **Ma**—median apophysis, **Mp**—median process, **Rd**—retrodorsal tibial apophysis, **Rl**—retrolateral tibial apophysis, **Rv**—retroventral tibial apophysis, **Ts**—tooth of the retrolateral tibial apophysis, **Vc**—retroventral crest.

Epigyne: **Cd**—copulatory duct, **Co**—copulatory opening, **Fo**—fovea, **Mr**—membranous part of the receptacle, **Rs**—sclerotized part of the receptacle, **Sl**—longitudinal scuta.

Depositories: **MHNG**—Muséum d’histoire naturelle, Genève, Switzerland (L. Monod); **ZMUT**—Zoological Museum of the University of Turku, Finland (V. Vahtera); **ZMMU**—Zoological Museum of the Moscow State University, Russia (K.G. Mikhailov); **ZMUU**—Zoological Museum of the Bursa Uludağ University, Turkiye (R.S. Kaya).

## ﻿Taxonomy

**Family Agelenidae C.L. Koch, 1837**

**Subfamily Ageleninae C.L. Koch, 1837**

### ﻿Tribe Agelenini

Taxon classificationAnimaliaAraneaeAgelenidae

C.L. Koch, 1837

B2060FA2-21AD-5881-B4A2-8C83082A1AD0

#### Comment.

For the diagnosis and composition, see Lehtinen (1967), Bolzern et al. (2013), and Zamani and Marusik (2020).

### ﻿ Persiscape caucasica

Taxon classificationAnimaliaAraneaeAgelenidae

(Guseinov, Marusik & Koponen, 2005)

E3E9BFD0-770A-5583-A767-9E659E0C78F0

Figs 1C , 2D, E

Agelescape caucasica Guseinov et al., 2005: 157, figs 9–12, 69–71, 105 (♀).
Persiscape caucasica : Zamani and Marusik 2020: 376, fig. 12M–O (♀).

#### Material.

Armenia: Kotayk Prov.: • 1 ♀ (ZMUT), env. of Geghadir, 40°09'N, 44°38'E, 15.05.2021 (Y.M. Marusik).

#### Comment.

The male of this species is currently unknown.

#### Distribution.

Previously known from Greece, Turkiye, Georgia, and Azerbaijan (WSC 2024). A new record for Armenia.

**Figure 1.**
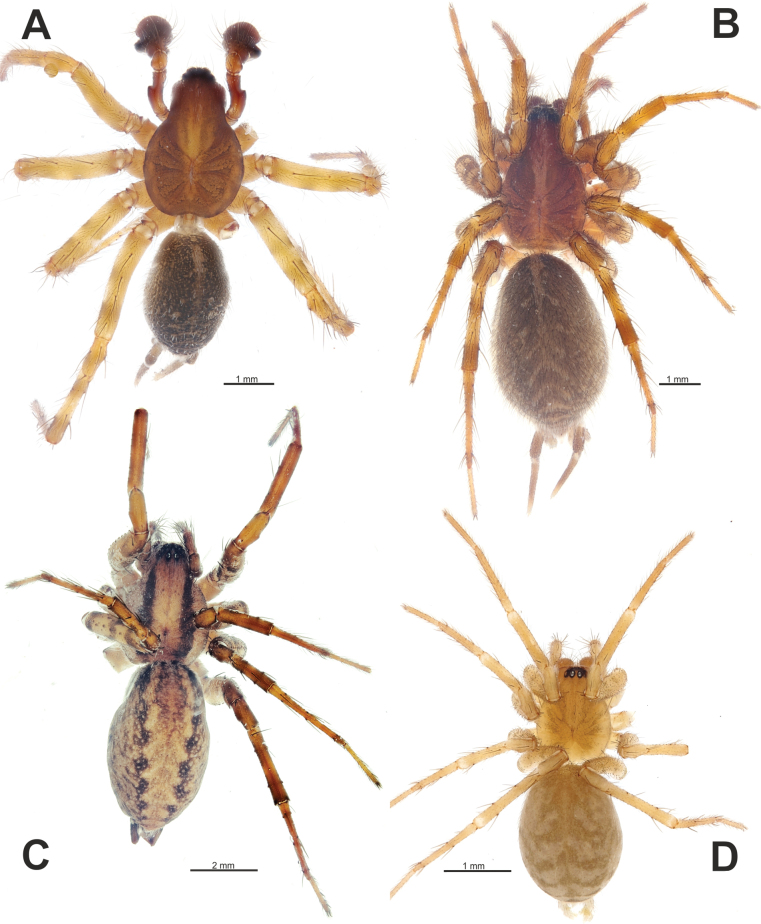
Habitus of *Maimunaantalyensis* sp. nov. (**A, B**), *Persiscapecaucasica* (**C**), and *Tegenariahamid* (**D**), dorsal view. **A** male **B–D** females.

**Figure 2.**
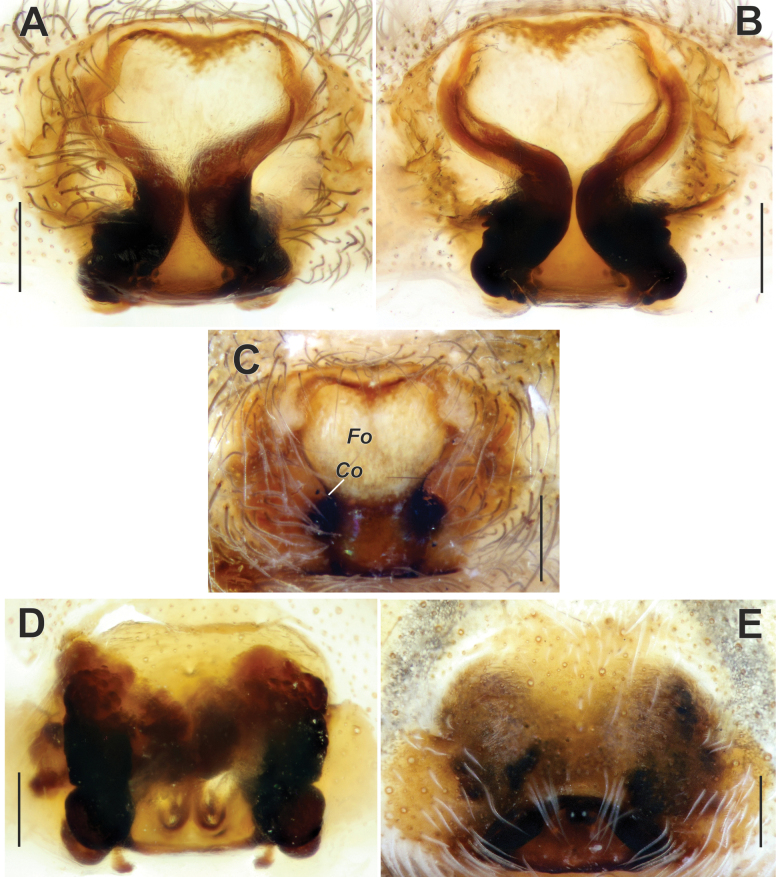
Epigyne of *Maimunaantalyensis* sp. nov. (**A–C**) and *Persiscapecaucasica* (**D, E**). **A** macerated, ventral view **B, D** vulva, dorsal view **C, E** intact, ventral view. Abbreviations: *Co* – copulatory opening, *Fo* – fovea. Scale bars: 0.2 mm.

### ﻿Tribe Textricini

Taxon classificationAnimaliaAraneaeAgelenidae

Lehtinen, 1967

8B3ABC85-3694-509F-8931-C7E3421960F7

#### Comment.

For the diagnosis and composition, see Kaya et al. (2023).

### ﻿ Maimuna antalyensis sp. nov.

Taxon classificationAnimaliaAraneaeAgelenidae

6D116E53-B5C0-538E-B0E1-62F745765614

https://zoobank.org/3A871504-2F88-4DF2-9053-90DFF4238122

**Figure 3.**
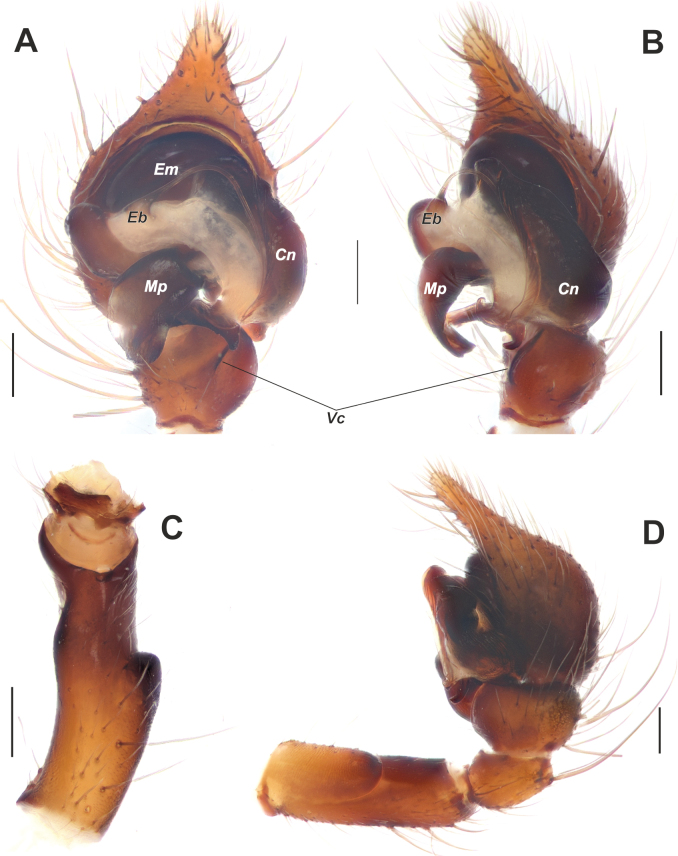
Male palp of *Maimunaantalyensis* sp. nov. **A** ventral view **B** retroventral view **C** femur, ventral view **D** retrolateral view. Abbreviations: *Eb* – base of the embolus, *Em* – embolus, *Cn* – conductor, *Mp* – median process, *Vc* – retroventral crest of the tibia. Scale bars: 0.2 mm.

**Figure 4.**
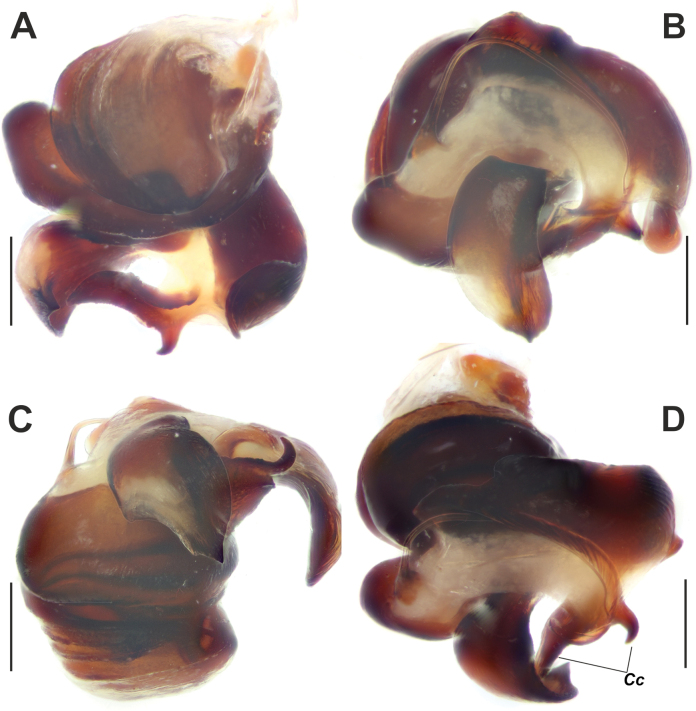
Dissected bulb of *Maimunaantalyensis* sp. nov. **A** proximal view **B** ventral view **C** proventral view **D** retroventral view. Abbreviation: *Cc* – claw-like projections of the conductor. Scale bars: 0.2 mm.

Figs 1A, B , 2A–C , 3A–D , 4A–D

#### Type material.

***Holotype*** • ♂ (ZMUT), Turkiye: Antalya Prov.: Alanya, env. Kestel, Dim Valley, 36°32'34.5"N, 32°06'17.5"E, 110 m, pine and oak forest, 2.01.2013 (Y.M. Marusik). ***Paratypes***: • 5 ♀ (ZMUT), same data as for the holotype; • 1 ♀ (ZMUT), Asmaca, 36°36'32.3"N, 32°03'12.4"E, 686 m, pine and oak forest, 3.01.2013 (Y.M. Marusik); • 2 ♀ (ZMUT), Elikesik rd., 36°33'55.6"N, 31°55'30.3"E, 24 m, maquis on S exposed slope, 8.01.2013 (Y.M. Marusik); • 2 ♀ (ZMUT), slopes of Alanya Castle, Damlataş side, 36°32'11.6"N, 31°59'30.3"E, 50 m, pine forest, under stones and in litter, 7.01.2013 (Y.M. Marusik).

#### Comparative material.

*Maimunavestita* (C.L. Koch, 1841): Turkiye: Bursa Prov.: • 1 ♀ (ZMUU), Bursa Uludağ University campus area, 30.11.1999 (R.S. Kaya); • 2 ♀ (ZMUU), same, 16.05.2000 (R.S. Kaya); • 1 ♀ (ZMUU), same, 3.03.2003 (R.S. Kaya); • 1 ♀ (ZMUU), same, 10.09.2005 (R.S. Kaya); • 1 ♂ 1 ♀ (ZMUU), same, 20.04.2012 (R.S. Kaya); • 1 ♂ 1 ♀ (ZMUU), same, 21.05.2012 (R.S. Kaya); • 5 ♀ (ZMUU), same, 4.05.2023 (R.S. Kaya); • 2 ♂ 3 ♀ (ZMUU), same, 4.01.2024 (R.S. Kaya); • 1 ♂ 2 ♀ (ZMUU), same, 11.07.2024 (R.S. Kaya); • 5 ♀ (ZMUU), Lake Uluabat, Halilbey Island, 8.04.2001 (R.S. Kaya); • 1 ♂ 3 ♀ (ZMUU), Lake Uluabat, Terzioğlu Island, 14.10.2004 (R.S. Kaya); • 5 ♀ (ZMUU), same, 25.04.2005 (R.S. Kaya); • 1 ♂ 2 ♀ (ZMUU), Lake Uluabat, Manastır Island, 28.09.2005 (R.S. Kaya); • 1 ♂ (ZMUU), same, 29.09.2005 (R.S. Kaya); • 2 ♂ 1 ♀ (ZMUU), Lake Uluabat, Halilbey Island, 15.12.2005 (R.S. Kaya); • 1 ♀ (ZMUU), Karacabey, Boğaz, 4.05.2005 (R.S. Kaya); • 3 ♀ (ZMUU), same, 8.08.2007 (R.S. Kaya); • 2 ♀ (ZMUU), same, 5.06.2018 (R.S. Kaya); • 2 ♀ (ZMUU), same, 11.06.2021 (R.S. Kaya); • 1 ♂ 1 ♀ (ZMUU), Kaplıkaya, 29.03.2007 (R.S. Kaya); • 2 ♂ 2 ♀ (ZMUU), same, 10.12.2008 (R.S. Kaya); • 1 ♀ (ZMUU), Görükle Vill., 20.04.2012 (R.S. Kaya); • 1 ♀ (ZMUU), Nilüfer Metro station, 26.05.2012 (R.S. Kaya); • 1 ♂ (ZMUU), Orhangazi Dist., 4.05.2014 (R.S. Kaya); Çanakkale Prov.: • 1 ♀ (ZMUU), Gökçeada Island, Lake salt area, 4.05.2004 (R.S. Kaya).

#### Diagnosis.

The new species is similar to *M.cariae* Brignoli, 1978 in the overall shape of its copulatory organs. The male differs by having a shorter tip of the cymbium (as long as the palpal tibia, vs longer), and by a different shape of the conductor and the median process (cf. Fig. 3B–D and Dimitrov 2022: fig. 26). The female of the new species has a hexagonal epigynal fovea, in contrast to the subtriangular fovea of *M.cariae* (cf. Fig. 2A, B and Dimitrov 2022: fig. 17).

#### Description.

**Male.** Habitus as in Fig. 1A. Total length 5.40. Carapace 2.60 long, 1.90 wide. Eye sizes: AME: 0.10, ALE: 0.13, PME: 0.18, PLE: 0.13. Carapace, sternum, labium, and maxillae pale brown; carapace with darker submedian bands; ocular region black. Legs yellowish brown, with annulations. Abdomen dorsally dark greyish with paler chevrons, pale greyish ventrally. Spinnerets pale greyish, darker basally. Measurements of legs: I: 5.81+missing Ta (1.74, 0.82, 1.43, 1.82, missing), II: 6.73 (1.77, 0.82, 1.37, 1.73, 1.04), III: 6.64 (1.74, 0.76, 1.34, 1.74, 1.06), IV: 8.53 (2.19, 0.85, 1.80, 2.55, 1.14).

Palp as in Figs 3A–D, 4A–D; femur modified – with retroventral bump in mid part, ~ 3× longer than wide, shorter than cymbium; dorsal length of patella same as in tibia; tibia ~ 1.4× wider than long (Fig. 3C, D), lacking prominent apophysis but with retroventral crest (*Vc*) (Fig. 3A, B); cymbium ~ 1.4× longer than wide, with tip ~ 1/3 of cymbial length; bulb as long as wide; conductor (*Cn*) massive, strongly sclerotized prolateral arm lacking, terminal part with two claw-like projections (*Cc*; Fig. 4D); median process (*Mp*) massive, roundly bent in lateral view (Fig. 4B, C); embolus (*Em*) filiform, weakly sclerotized, with small base (*Eb*).

**Figure 5.**
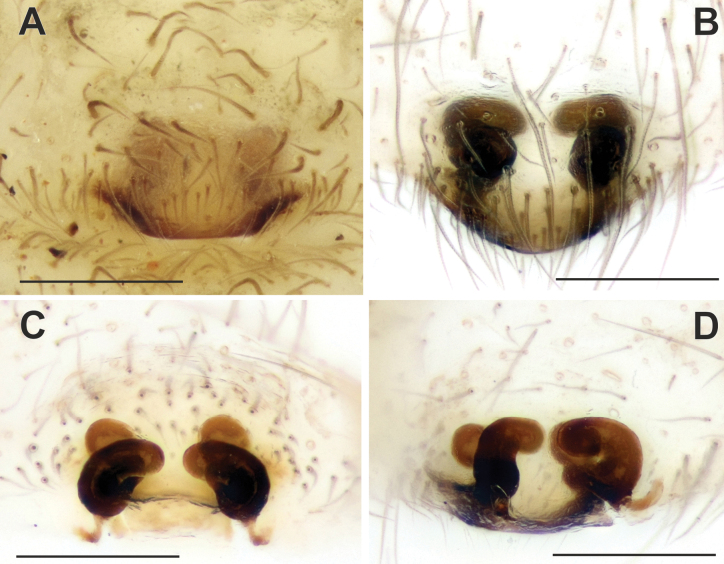
Epigyne of *Tegenariahamid*. **A** intact, ventral view **B** macerated, anteroventral view **C** vulva, dorsal view **D** vulva, anterodorsal view. Scale bars: 0.2 mm.

**Female.** Habitus as in Fig. 1B. Total length 7.50. Carapace 3.28 long, 2.07 wide. Eye sizes: AME: 0.12, ALE: 0.17, PME: 0.20, PLE: 0.15. Coloration as in male. Measurements of legs: I: 7.25 (1.94, 1.02, 1.44, 1.76, 1.09), II: 7.24 (1.85, 1.05, 1.47, 1.73, 1.14), III: 7.37 (2.00, 0.96, 1.42, 1.94, 1.05), IV: 9.58 (2.45, 1.11, 2.13, 2.66, 1.23).

Epigyne as in Fig. 2A–C; epigynal plate slightly wider than long; fovea (*Fo*) hexagonal, approximately as long as wide, located anteriorly (Fig. 2C); copulatory ducts gradually turning to receptacles and approximately as wide as receptacles; copulatory ducts converging and contiguous, receptacles diverging (Fig. 2A, B).

#### Comment.

In the examined comparative female specimens of *M.vestita*, we observed noticeable variation in both body size and epigyne morphology. In particular, in smaller individuals, the shape of the epigynal fovea can vary significantly. A similar pattern is observed in *M.antalyensis* sp. nov., where smaller females also exhibit variation in the shape of the epigynal fovea, ranging from hexagonal to nearly circular in some specimens.

#### Note.

For comments on the homology of the structure referred to here as the “median process,” see Kaya et al. (2023).

#### Distribution.

Known from the listed localities in Antalya Province, southwestern Turkiye.

#### Etymology.

The specific epithet refers to the type locality of the species in Antalya, Turkiye.

### ﻿Tribe Tegenariini

Taxon classificationAnimaliaAraneaeAgelenidae

Lehtinen, 1967

8DA84A35-41F7-5909-A22F-5AF0554E9CEC

#### Comment.

For the diagnosis and composition, see Bolzern et al. (2010).

### ﻿ Tegenaria anhela

Taxon classificationAnimaliaAraneaeAgelenidae

Brignoli, 1972

4F95A87E-2570-5DCE-B7E5-A13AD79D1690

**Figure 6.**
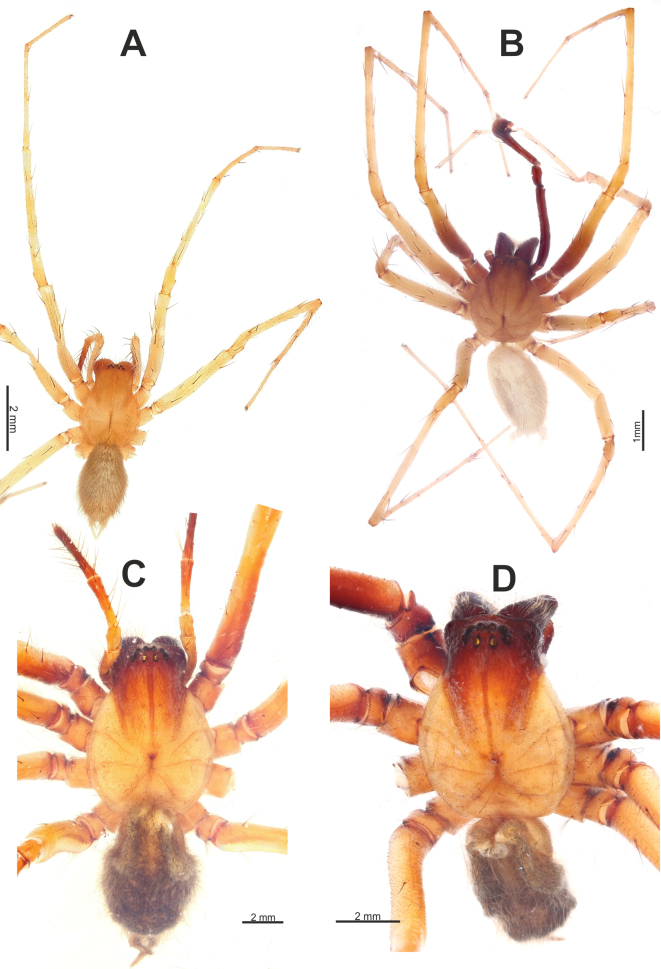
Habitus of *Tegenariaanhela* (**A, B**) and *Tegenariaballarini* sp. nov. (**C, D**). **A, C** females **B, D** males.

**Figure 7.**
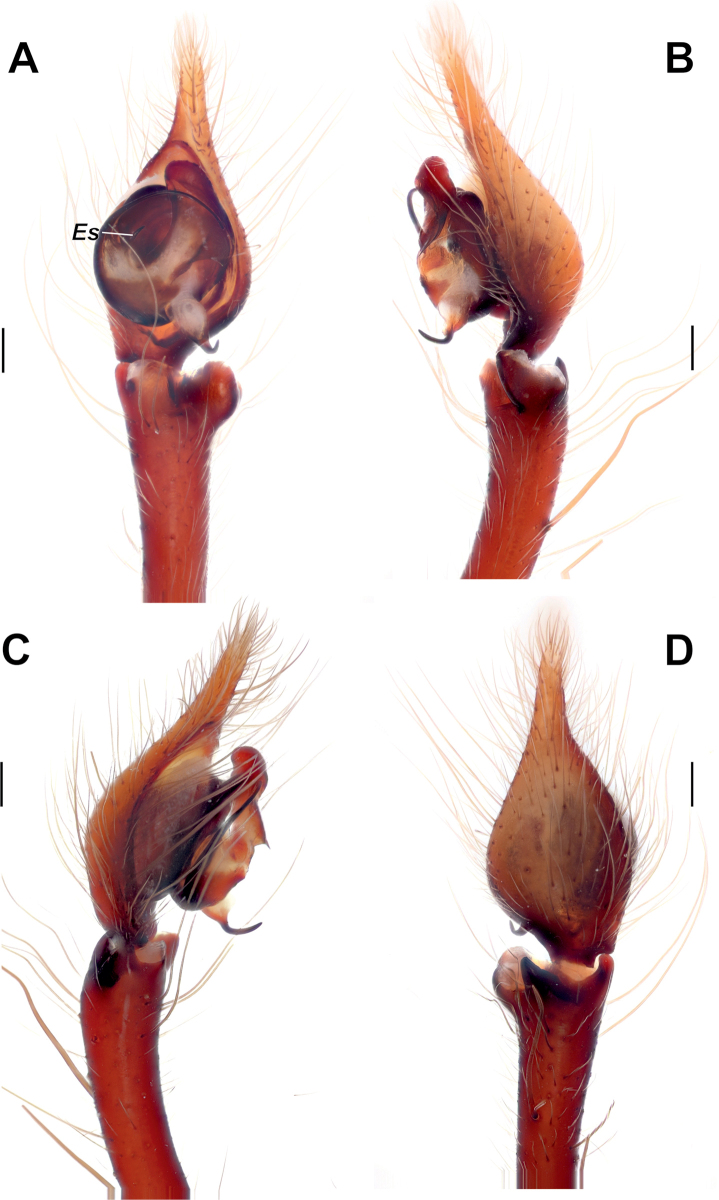
Male palp of *Tegenariaanhela*. **A** ventral view **B** retrolateral view **C** prolateral view **D** dorsal view. Abbreviation: *Es* – embolic spine. Scale bars: 0.2 mm.

**Figure 8.**
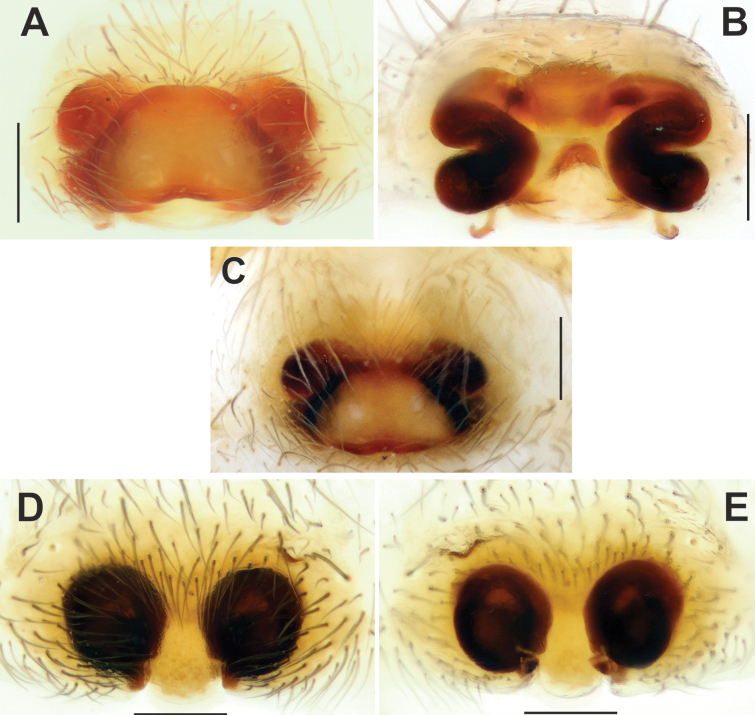
Epigyne of *Tegenariaanhela* (**A–C**) and *T.averni* (**D, E**). **A, D** macerated, ventral view **B, E** vulva, dorsal view **C** intact, ventral view. Scale bars: 0.2 mm.

Figs 6A, B , 7A–D , 8A–C , 20B

Tegenaria anhela Brignoli, 1972: 173, figs 24–27 (♂♀).
Malthonica anchela : Guseinov et al. 2005: 164 (lapsus).
Tegenaria anhela : Bolzern et al. 2013: 846.

#### Material.

Turkiye: Antalya Prov.: • 1 ♂ 1 ♀ (ZMUT), Döşemealtı, 37°01'N, 30°36'E, 4.08.2009 (R.S. Kaya, C. Kaya); • 1 ♂ 1 ♀ (ZMUU). same data; • 2 ♀ (ZMUU), Döşemealtı, Karain Cave (R.S. Kaya, C. Kaya).

#### Comments.

*Tegenariaanhela* was described based on material collected from Karain Cave in Antalya (Brignoli 1972). Later, it was recorded from Mustan Ini Cave, which is located near the type locality (Brignoli 1978a). Although the original description includes informative illustrations, it lacks detailed explanations of the palpal structures. During the examination of our material, we observed a unique structure, which we term the “embolic spine” (*Es*; Fig. 7A). This structure, the homology of which remains unclear, is situated between the base of the embolus and the conductor, and it has not been reported in any other *Tegenaria* species to date.

#### Distribution.

Known only from Antalya Province, southwestern Turkiye.

### ﻿ Tegenaria averni

Taxon classificationAnimaliaAraneaeAgelenidae

Brignoli, 1978

3B854A84-7F4D-52D5-B235-89302FC9E077

Figs 8D, E , 13A–D

Tegenaria averni Brignoli, 1978a: 50, fig. 10 (♀).
Tegenaria lazarovi Dimitrov, 2020: 48, figs 1–12 (♂♀). Syn. nov.

#### Material.

Turkiye: Mersin Prov.: • 2 ♂ 2 ♀ (ZMUT), Silifke, Cennet Cave, 36°26'12"N, 34°06'22"E, 20.09.2010 (Y.M. Marusik).

#### Comments.

*Tegenariaaverni* was described based on a single female from Cennet Cave in Mersin (Brignoli 1978a). In his review of the *ariadnae* species-group, Dimitrov (2020) described *T.lazarovi* based on material of both sexes collected in a cave in Mersin. Although the type locality of *T.lazarovi* is only 65 km away from that of *T.averni*, and both are in the same provincial district, *T.averni* is not mentioned in Dimitrov (2020) at all, likely due to a lack of males. Collection of material of both sexes at the type locality of *T.averni* revealed that these two populations are conspecific. Therefore, *T.lazarovi* syn. nov. is proposed as a junior synonym of *T.averni*.

#### Distribution.

Known only from two caves in Mersin Province, southern Turkiye.

### ﻿ Tegenaria ballarini sp. nov.

Taxon classificationAnimaliaAraneaeAgelenidae

74671553-5905-5A02-BAD6-1AF3751E4036

https://zoobank.org/BB2AE92D-4A0E-4BFD-9FC5-8F2434070636

**Figure 9.**
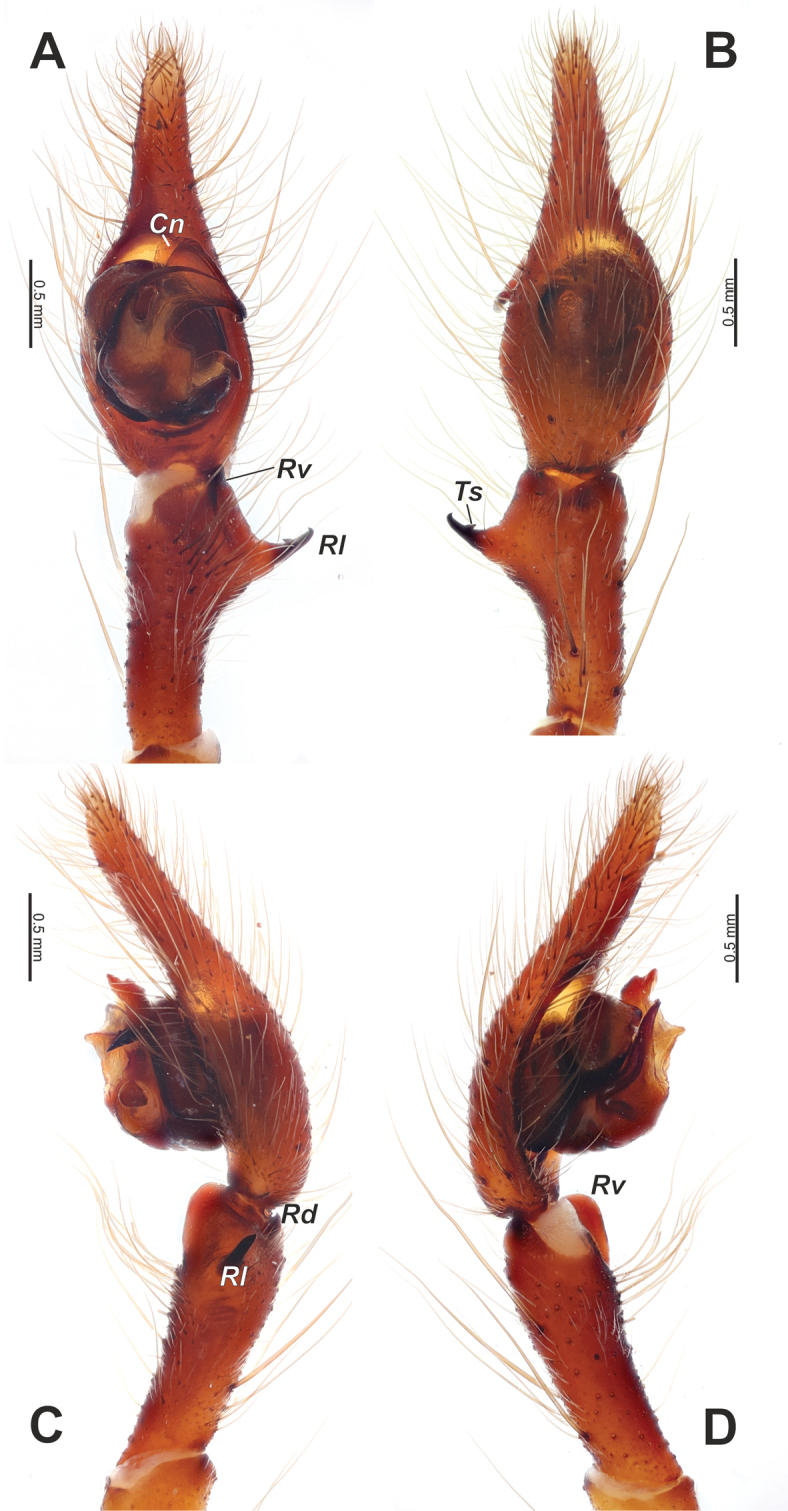
Male palp of *Tegenariaballarini* sp. nov. **A** ventral view **B** dorsal view **C** retrolateral view **D** prolateral view. Abbreviations: *Cn* – conductor, *Rd* – retrodorsal apophysis, *Rl* – retrolateral apophysis, *Rv* – retroventral apophysis, *Ts* – small tooth of the retrolateral apophysis.

**Figure 10.**
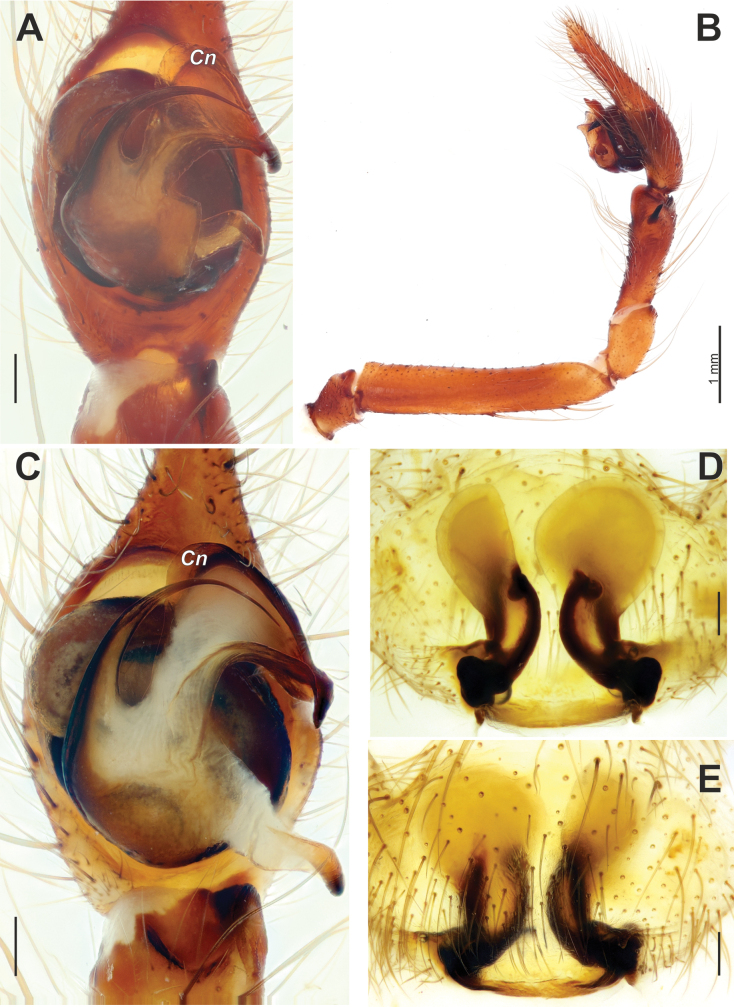
Copulatory organs of *Tegenariaballarini* sp. nov. (**A, B**) and *T.vankeerorum* (**C–E**). **A, C** palp, ventral view **B** full palp, retrolateral view **D** vulva, dorsal view **E** macerated epigyne, ventral view. Abbreviation: *Cn* – conductor. Scale bars: 0.2 mm, unless otherwise indicated.

Figs 6C, D , 9A–D , 10A, B , 12A–C

#### Type material.

***Holotype*** • ♂ (ZMUU), Turkiye: Antalya Prov.: Bozyaka, Köprülü Canyon National Park, 37°11'51"N, 31°11'03"E, 243 m, 15.05.2008 (R.S. Kaya). ***Paratypes***: • 1 ♂ 2 ♀ (ZMUT), same data as for the holotype; • 7 ♀ (ZMUU), same data as for the holotype.

#### Comparative material.

**Figure 11.**
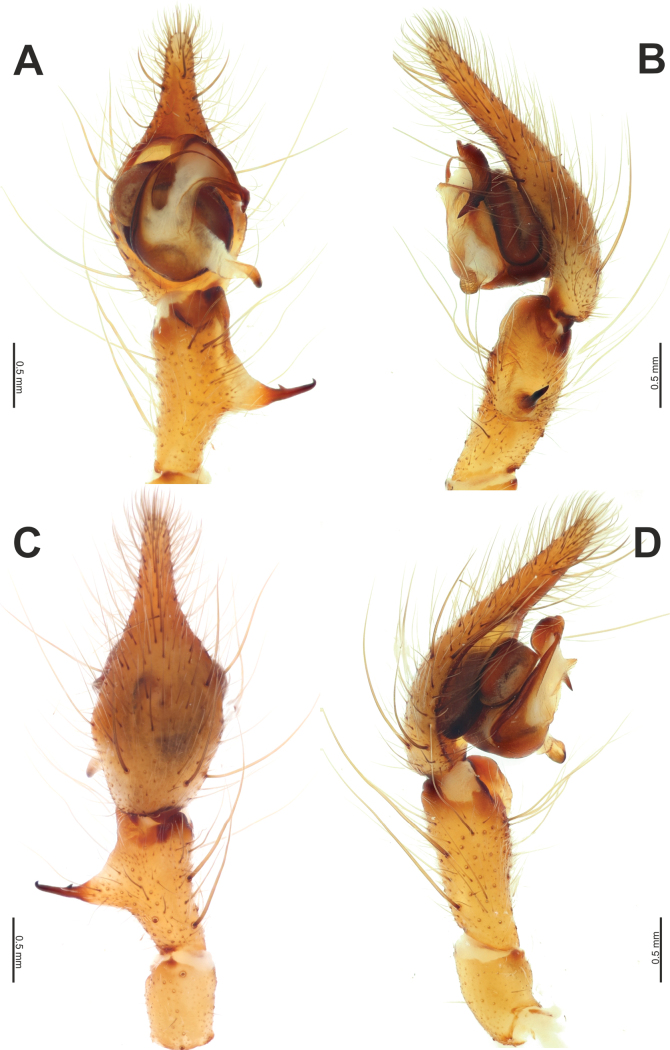
Male palp of *Tegenariavankeerorum*. **A** ventral view **B** retrolateral view **C** dorsal view **D** prolateral view.

*Tegenariavankeerorum* Bolzern, Burckhardt & Hänggi, 2013 (Figs 10C, D, 11A–D): Turkiye: Muğla Prov.: • 1 ♂ 1 ♀ (ZMUT), Yatağan, Orman İşletme, 37°20'N, 28°08'E, 18.05.2011 (R.S. Kaya).

#### Diagnosis.

**Figure 12.**
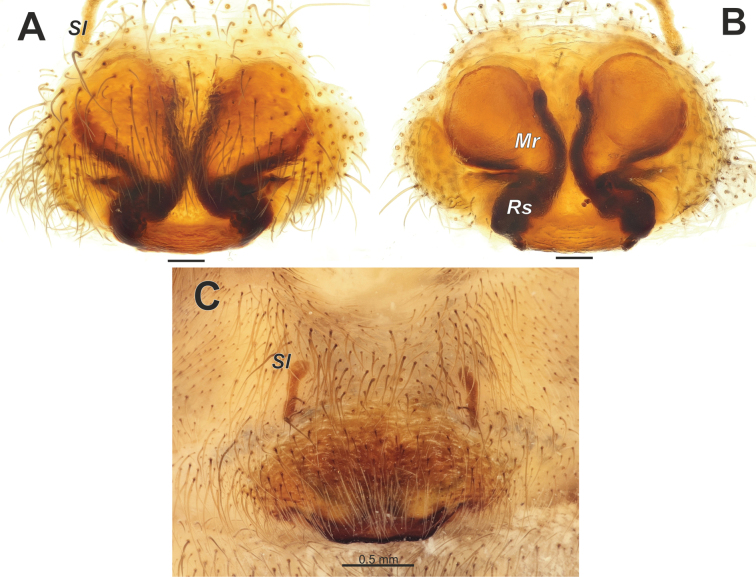
Epigyne of *Tegenariaballarini* sp. nov. **A** macerated, ventral view **B** vulva, dorsal view **C** intact, ventral view. Abbreviations: *Mr* – membranous part of the receptacle, *Rs* – sclerotized part of the receptacle, *Sl* – longitudinal scutum of the epigynal plate. Scale bars: 0.2 mm, unless otherwise indicated.

The new species is closely related to *T.vankeerorum* and has very similar copulatory organs, especially the male palp. The male of *T.ballarini* sp. nov. differs from the similar species by having relatively longer palpal tibia and a retrolateral apophysis (*Rl*) located in the distal half of the tibia, rather than at the midpoint (cf. Figs 9C, 10B, 11B). The female of the new species differs from all other species of *Tegenaria* by having a pair of longitudinal scuta (*Sl*) anterior to the epigynal plate and a straight posterior margin of the epigyne (Fig. 12C). Additionally, the vulva of the new species differs from that of *T.vankeerorum* by having relatively longer copulatory ducts that almost reach the anterior margin of the receptacle (vs reaching only the mid part of the receptacle; cf. Figs 12A, B, 10D, E).

#### Description.

**Male.** Habitus as in Fig. 6D. Total length 10.75. Carapace 6.75 long, 4.65 wide. Eye sizes: AME: 0.25, ALE: 0.25, PME: 0.22, PLE: 0.25. Pars cephalica, sternum, labium, maxillae, and Fe I brown; pars thoracica and remaining leg segments yellowish brown; chelicerae dark reddish brown. Legs without annulations. Abdomen dark grey, without patterns. Spinnerets uniformly greyish brown. Measurements of legs: I: 48.87+missing Ta (14.28, 2.82, 14.60, 17.17, missing), II: 44.00 (12.00, 2.72, 11.50, 14.28, 3.50), III: 36.83 (9.98, 2.45, 8.65, 12.65, 3.10), IV: 44.36 (11.56, 2.50, 10.88, 15.82, 3.60).

Palp as in Figs 9A–D, 10A, B; femur 5× longer than wide and ~ 1.8× longer than tibia; patella ~ 1.9× longer than wide; tibia long, ~ 3.5× longer than wide, with three apophyses: retrolateral (*Rl*), retroventral (*Rv*) and retrodorsal (*Rd*) (Fig. 9A–C); retrolateral apophysis located in distal 1/3 of tibia, spine-like, directed antero-retrolaterally, with small tooth (*Ts*) (Fig. 9B); cymbium long, 2.6× longer than wide, tip as long as bulb (Fig. 10B); bulb as long as wide (accounting conductor), tegulum oval, bent prolaterally, and distal part extending embolus; conductor (*Cn*) with rounded distal arm and gradually tapering proximal arm with claw-like tip, terminated at ~ 3 o’clock position; embolus originates at 9 o’clock position, thick basally, roundly bent (Fig. 10A).

**Female.** Habitus as in Fig. 6C. Total length 14.15. Carapace 8.20 long, 6.15 wide. Eye sizes: AME: 0.25, ALE: 0.27, PME: 0.28, PLE: 0.31. Coloration as in male. Measurements of legs: I: 42.17 (11.61, 3.40, 11.17, 12.34, 3.65), II: 37.29 (10.48, 2.98, 9.36, 11.12, 3.35), III: 33.05 (9.40, 2.75, 7.70, 10.20, 3.00), IV: Fe: 11.03, Pa: 2.98, Ti: 10.03, remaining segments missing.

Epigyne as in Fig. 12A–C; epigynal plate > 2× wider than long, with straight and heavily sclerotized posterior margin; area anteriorly from plate with pair of elongate scuta (*Sl*) (Fig. 12C); receptacles subdivided into two parts, anterior pear-shaped membranous part (*Mr*) and heavily sclerotized posterior parts (*Rs*) (Fig. 12B); copulatory ducts well sclerotized, thin, contiguous, terminating near anterior edges of receptacles.

**Figure 13.**
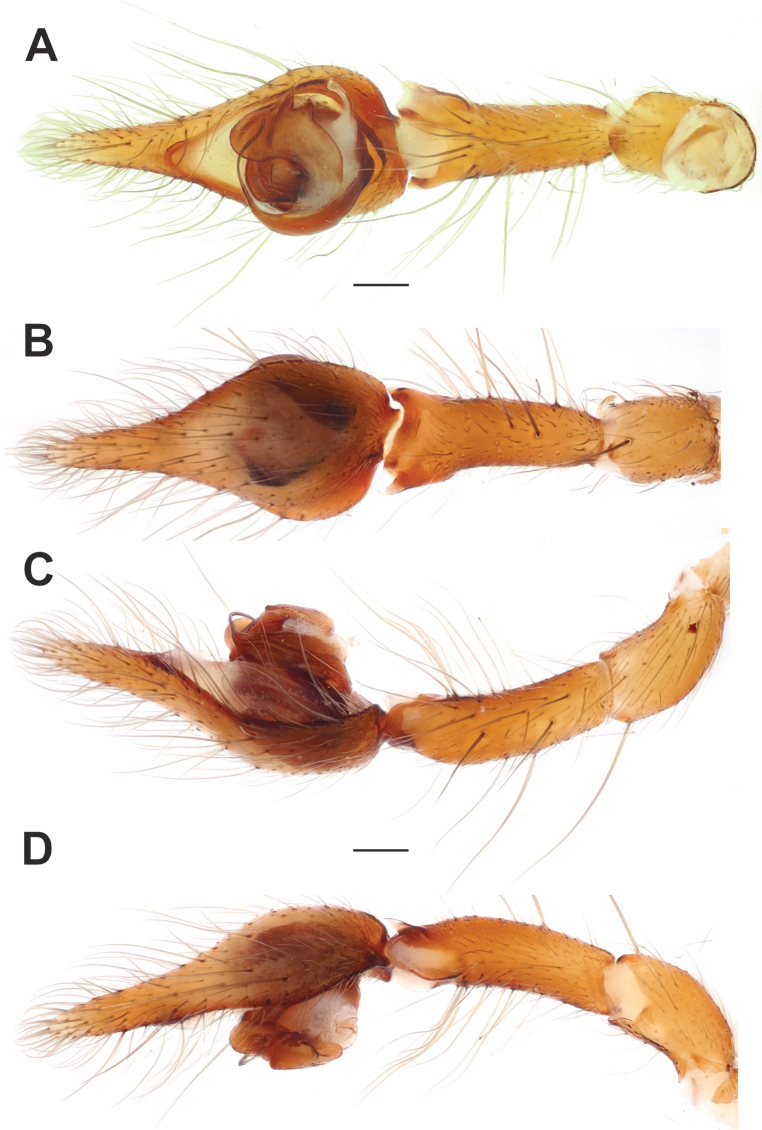
Male palp of *Tegenariaaverni*. **A** ventral view **B** dorsal view **C** prolateral view **D** retrolateral view. Scale bars: 0.2 mm.

#### Distribution.

Known only from the type locality in Antalya Province, southwestern Turkiye.

#### Etymology.

The new species is named in honor of our colleague Francesco Ballarin (Tokyo, Japan), in recognition of his assistance to the second author during her visit to the Brignoli collection in Verona, Italy.

### ﻿ Tegenaria beyazcika sp. nov.

Taxon classificationAnimaliaAraneaeAgelenidae

6282C83F-B87B-55A5-86A8-CD282839F154

https://zoobank.org/2EEF98AE-7348-4DEA-AE26-AE050EFBA76A

Figs 14A–D , 18D

#### Type material.

***Holotype*** • ♂ (ZMUT), Turkiye: Antalya Prov.: Alanya, env. Kestel, Dim Valley, 36°32'34.5"N, 32°06'17.5"E, 110 m, pine and oak forest, 2.01.2013 (Y.M. Marusik). ***Paratypes***: • 4 ♂ (ZMUT), same data as for the holotype.

#### Diagnosis.

**Figure 14.**
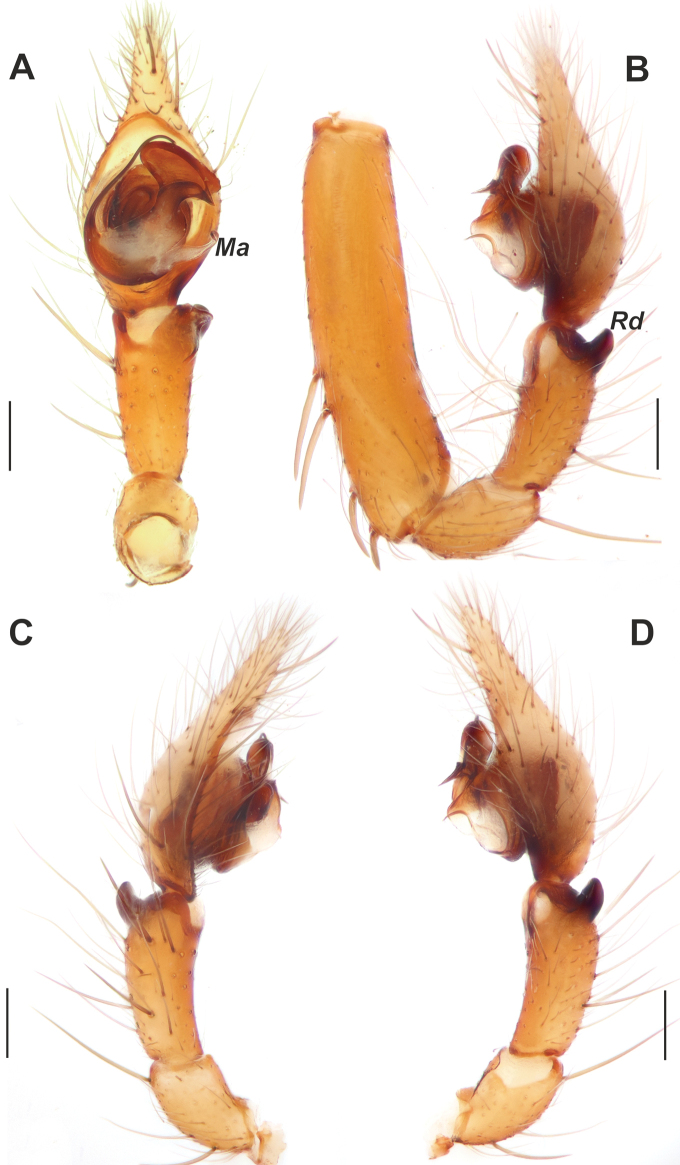
Male palp of *Tegenariabeyazcika* sp. nov. **A** ventral view **B** full palp, retrolateral view **C** prolateral view **D** retrolateral view. Abbreviations: *Ma* – median apophysis, *Rd* – retrodorsal apophysis. Scale bars: 0.2 mm.

The new species belongs to the *ariadnae* species-group and is most similar to *T.averni*. The male of the new species differs from that of *T.averni* by having thickened male palpal femur with four strong dorsal spines (Fig. 14B), an almost straight embolus on the prolateral half (vs roundly bent), a relatively shorter tibia with a length/width ratio of 2.5 (vs 2.9), and a conductor with subequal arms (vs a distal arm that is longer than the proximal arm; cf. Figs 14A, 13A).

#### Description.

**Male.** Habitus as in Fig. 18D. Total length 4.00. Carapace 1.95 long, 1.55 wide. Eye sizes: AME: 0.04, ALE: 0.07, PME: 0.06, PLE: 0.07. Pars cephalica, chelicerae, labium, maxillae, and Fe I and II pale brown, Fe II paler than I; pars thoracica, sternum, and remaining leg segments pale brown. Legs without annulations. Fe I, and to lesser degree Fe II, with ventral coating of long setae. Abdomen pale beige, without patterns. Spinnerets uniformly pale beige. Measurements of legs: I: 9.00 (2.36, 0.78, 2.24, 2.24, 1.38), II: 7.95 (2.16, 0.74, 1.85, 1.93, 1.27), III: 7.39 (1.93, 0.67, 1.65, 2.00, 1.14), IV: 9.78 (2.55, 0.75, 2.36, 2.74, 1.38).

Palp as in Fig. 14A–D; femur 4× longer than wide, longer than cymbium, 1.5× wider than tibia, with 4 strong spines in distal 1/2 (Fig. 14B); patella 2× longer than wide; tibia 2.25× longer than wide, with retroventral (*Rv*) and retrodorsal (*Rd*) apophyses (Fig. 14B); cymbium > 2× longer than wide, tip approximately as long as cymbium wide, with two strong macrosetae (= spines) on retrolateral 1/2; bulb as long as wide; median apophysis (*Ma*) long, approximately as long as width of tibia, originating at ~ 5 o’clock position (Fig. 14A); conductor fungiform, with both arms of equal length and width; embolus originating at ~ 8 o’clock position, straight in prolateral 1/2 of bulb and strongly roundly bent proximally at retrolateral side.

**Female.** Unknown.

#### Comments.

Although the specimens of both *T.hamid* and *T.beyazcika* sp. nov. (known only from females and males, respectively) were collected from the same locality, we consider them to belong to different species due to noticeable differences in size and coloration. Additionally, *T.hamid* has a different conformation of the copulatory organs compared to those of the species in the *ariadnae* group, thus belonging to a different species-group than *T.beyazcika* sp. nov. Given the pale coloration of this species, the relatively elongated legs, and the dense ventral coating of long setae on femora I and II, it seems that the collection locality mentioned on the label is slightly off. It is more likely that the species was collected from a cave, such as the nearby Dim Cave.

#### Distribution.

Known only from the type locality in Antalya Province, southwestern Turkiye.

#### Etymology.

The specific epithet is derived from the Turkish word "beyaz", meaning pale, combined with the suffix -cik, meaning little. This refers to the relatively small size and pale coloration of this species.

### ﻿ Tegenaria chumachenkoi

Taxon classificationAnimaliaAraneaeAgelenidae

Kovblyuk & Ponomarev, 2008

2E522131-4D46-596A-A844-82DD8581EA45

Fig. 15A–D

Tegenaria chumachenkoi Kovblyuk & Ponomarev, 2008: 147, figs 18–21 (♀).
Tegenaria chumachenkoi : Ponomarev and Shmatko 2022: 212, figs 5–10 (♂♀). – Seropian et al. 2023: 147, suppl.: 5 fig. (♂).

#### Material.

Turkiye: Artvin Prov.: • 1 ♂ (ZMUT), Şavşat Dist., env. of Meydancık Town, Erikli Vill., 41°27'13.1"N, 42°13'23.8"E, 1141 m, 12.06.2009 (Y.M. Marusik).

#### Distribution.

Previously known from Azerbaijan, Georgia, and northern Caucasus (Ponomarev and Shmatko 2022). A new record for Turkiye.

**Figure 15.**
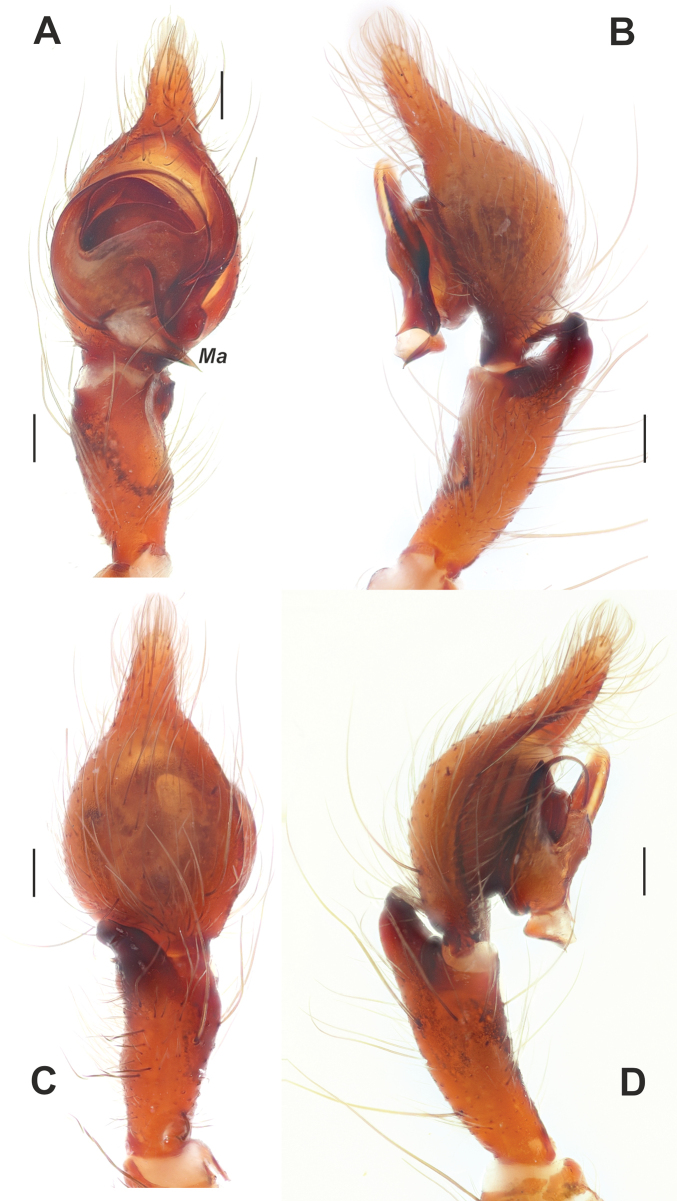
Male palp of *Tegenariachumachenkoi*. **A** ventral view **B** retrolateral view **C** dorsal view **D** prolateral view. Abbreviation: *Ma* – median apophysis. Scale bars: 0.2 mm.

### ﻿ Tegenaria dalmatica

Taxon classificationAnimaliaAraneaeAgelenidae

Kulczyński, 1906

C2D8E2B5-9237-56AC-9BB3-417FC0082C14

Figs 16A–C , 18C

Malthonica dalmatica : Kovblyuk and Nadolny 2007: 19, figs 1–10 (♂♀).
Tegenaria dalmatica : Bolzern et al. 2013: 793, figs 1G, H, 2A, B, D, 15K, L, O, Q (♂♀).

#### Note.

For a full list of 14 taxonomic entries, see WSC (2024).

#### Material.

Turkiye: Izmir Prov.: • 6 ♀ (ZMUT), Kemalpaşa, Vişneli Vill., Fetrek-2 Cave, 38°20'N, 27°25'E, 311 m, 5.06.2009 (Y.M. Marusik); Bursa Prov.: • 1 ♂ (ZMUU), Lake Uluabat, Terzioğlu Island, 21.11.2003 (R.S. Kaya); • 1 ♀ (ZMUU), Lake İznik, Göllüce Vill., 14.10.2016, (R.S. Kaya).

**Figure 16.**
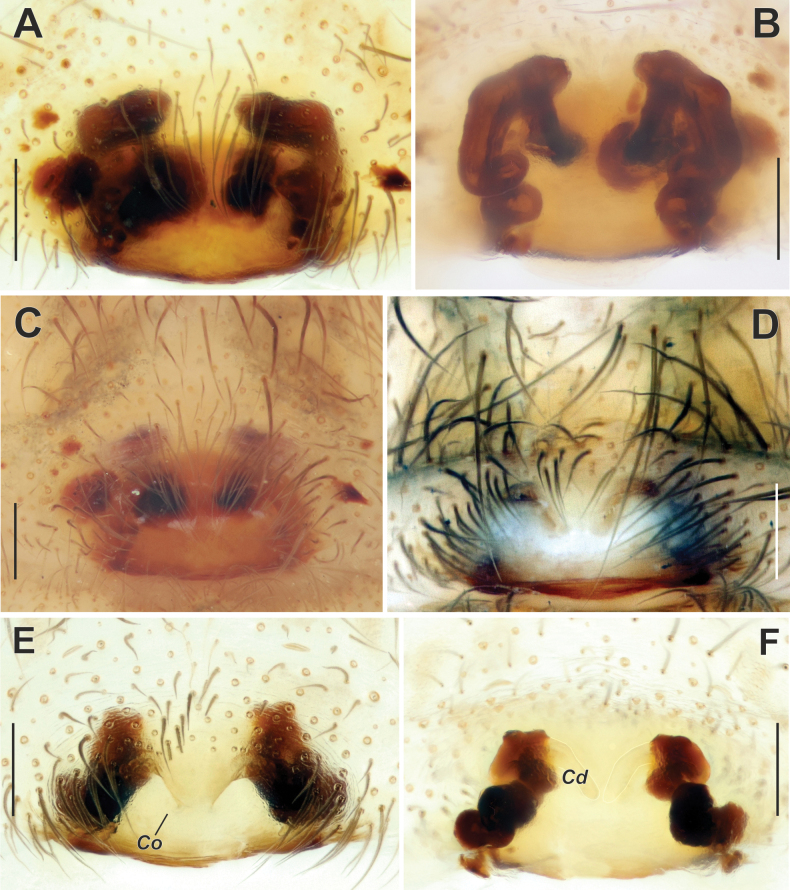
Epigyne of *Tegenariadalmatica* (**A–C**) and *T.egrisiana* sp. nov. (**D–F**). **A, E** macerated, ventral view **B, F** vulva, dorsal view **C, D** intact, ventral view. Abbreviations: *Cd* – copulatory duct, *Co* – copulatory opening. Scale bars: 0.2 mm.

#### Comment.

The only previous record of this species from Turkiye was by Bolzern et al. (2013), although lacking further locality data.

#### Distribution.

From Iberian Peninsula to Turkiye, south to northern Africa (Nentwig et al. 2024; WSC 2024).

### ﻿ Tegenaria egrisiana sp. nov.

Taxon classificationAnimaliaAraneaeAgelenidae

9008C635-326A-558A-A461-B8C347C30196

https://zoobank.org/45CD9A9F-8B0B-47DD-B0A7-145458C6B6BE

**Figure 17.**
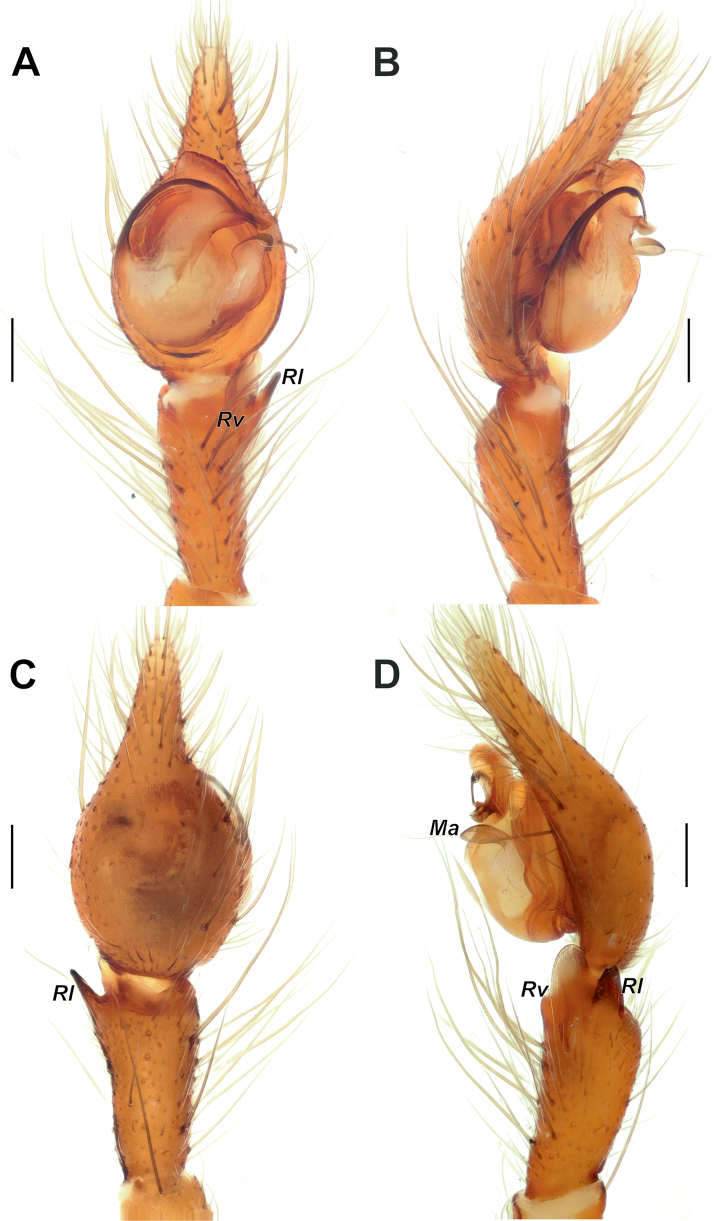
Male palp of *Tegenariaegrisiana* sp. nov. **A** ventral view **B** prolateral view **C** dorsal view **D** retrolateral view. Abbreviations: *Ma* – median apophysis, *Rl* – retrolateral apophysis, *Rv* – retroventral apophysis. Scale bars: 0.2 mm.

**Figure 18.**
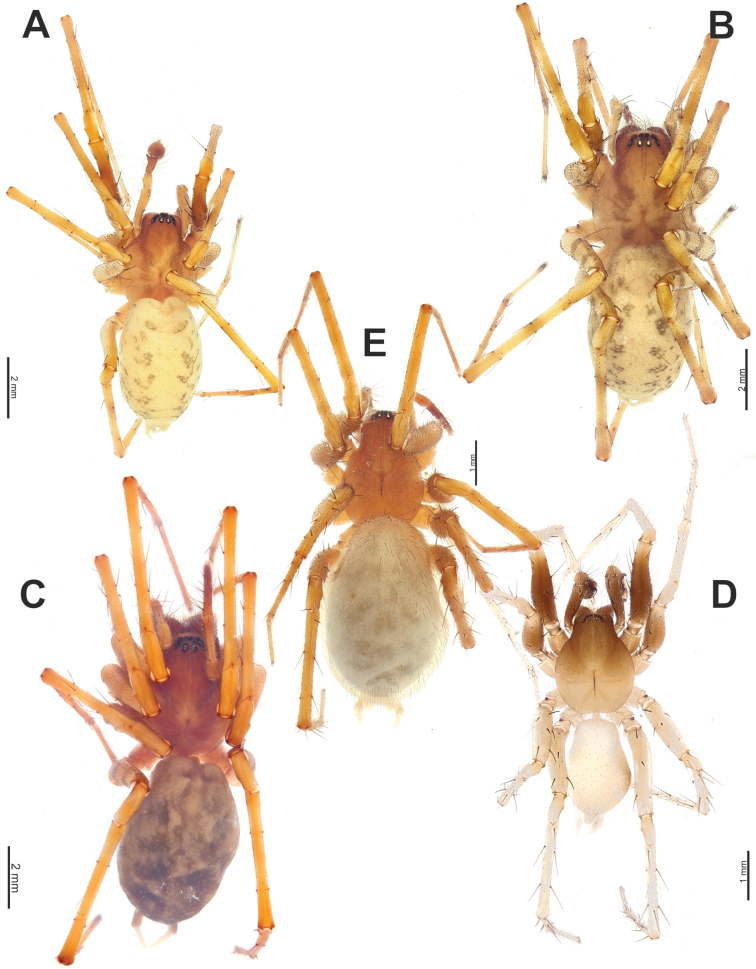
Habitus of *Tegenariaegrisiana* sp. nov. (**A, B**), *T.dalmatica* (**C**), *T.beyazcika* sp. nov. (**D**), and *T.tekke* (**E**), dorsal view. **A, D** males **B, C, E** females.

Figs 16D–F , 17A–D , 18A, B , 20C

#### Type material.

***Holotype*** • ♂ (ZMMU), Georgia: Imereti Prov.: cave between Gumbrini and Khamali, 42°18'56.4"N, 42°38'09.4"E, 161 m, 19.07.2012 (Y.M. Marusik). ***Paratypes***: • 1 ♂ 1 ♀ (ZMUT), 2 ♀ (ZMMU), same data as for the holotype.

#### Diagnosis.

*Tegenariaegrisiana* sp. nov. is very similar to *T.pallens* Zamani & Marusik, 2023 from Iran in the overall shape of the copulatory organs. However, the male differs from *T.pallens* in the shorter tip of the cymbium, ~ 0.7 the length of the palpal tibia (Fig. 17A–D, 20C; vs as long as the palpal tibia), the blunt tip of the conductor (vs pointed and curved; Zamani et al. 2023: fig. 2A), the embolus base positioned at the 9:00 o’clock position (vs 8:30 o’clock), the tip of the embolus terminating at ~ 2:00 o’clock position (Fig. 17A; vs 1:00 o’clock), and the median apophysis (*Ma*) with a different shape. The female of the new species differs from that of *T.pallens* in the epigynal plate nearly twice as wide as it is long (vs > 3× wider than long; cf. Fig. 16D and Zamani et al. 2023: fig. 3C), in having a distinct median plate (vs absent), and a small rectangular fovea (vs oval; cf. Fig. 16D, E and Zamani et al. 2023: fig. 3A, B).

**Figure 23.**
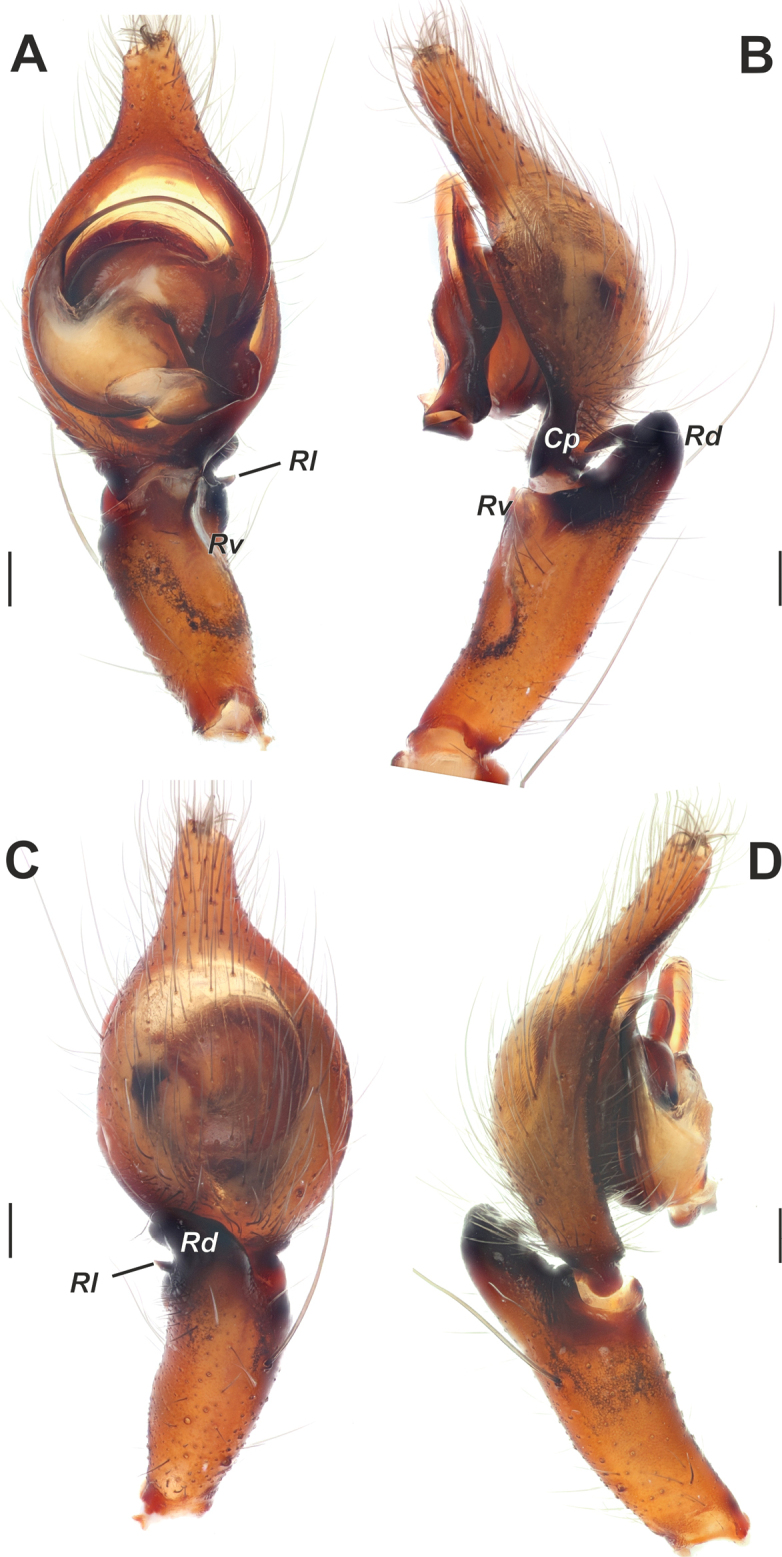
Male palp of *Tegenariahoeferi* sp. nov. **A** ventral view **B** retrolateral view **C** dorsal view **D** prolateral view. Abbreviations: *Cp* – basal process of the cymbium, *Rd* – retrodorsal apophysis, *Rl* – retrolateral apophysis, *Rv* – retroventral apophysis. Scale bars: 0.2 mm.

**Figure 24.**
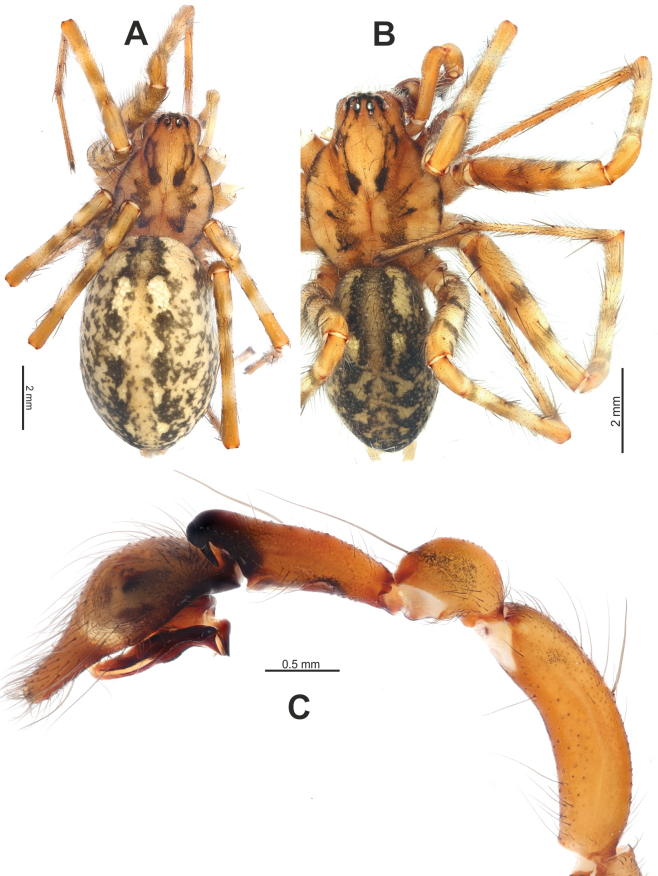
*Tegenariahoeferi* sp. nov. **A, B** habitus, dorsal view **C** full palp, retrolateral view. **A** female **B, C** male.

#### Description.

**Male.** Habitus as in Fig. 18A. Total length 7.10. Carapace 3.23 long, 2.37 wide. Eye sizes: AME: 0.12, ALE: 0.17, PME: 0.14, PLE: 0.18. Carapace, labium, and maxillae pale brown; carapace with darker submedian bands; chelicerae reddish brown; sternum greyish brown, with yellow median band and six spots. Legs pale brown, with very faint annulations; Fe with long ventral setae at basal 1/2. Abdomen pale beige, with greyish dots, patches, and stripes. Spinnerets uniformly pale beige. Measurements of legs: I: 22.92 (6.12, 1.44, 6.26, 6.50, 2.60), II: 19.02 (5.05, 1.28, 4.96, 5.50, 2.23), III: 16.90 (4.63, 1.15, 4.15, 5.15, 1.82), IV: 20.79 (5.50, 1.28, 5.24, 6.65, 2.12).

Palp as in Fig. 17A–D; femur longer than patella+tibia; femur ~ 2.2× longer than tibia (Fig. 20C); patella 2× longer than wide; cymbium ~ 1.8× longer than tibia; tibia ~ 2× longer than wide, with two apophyses: large and membranous retroventral apophysis (*Rv*) and conical retrolateral apophysis (*Rl*) with a notched blunt tip (Figs 17D, 20C); retrolateral apophysis shorter than ventrolateral one; cymbium 2× longer than wide; bulb longer than wide; median apophysis (*Ma*) large and wide, originating at ~ 4 o’clock position; conductor as long as wide, with a spatula-like tip; embolus filiform, roundly bent, originating at ~ 9:00 o’clock position (Fig. 17A).

**Female.** Habitus as in Fig. 18B. Total length 8.68. Carapace 4.25 long, 2.95 wide. Eye sizes: AME: 0.12, ALE: 0.20, PME: 0.18, PLE: 0.20. Coloration as in male. Measurements of legs: I: 22.80 (6.13, 1.65, 6.00, 6.30, 2.72), II: 19.81 (5.53, 1.57, 4.96, 5.47, 2.28), III: 18.03 (5.07, 1.44, 4.32, 5.22, 1.98), IV: 22.57 (6.18, 1.47, 5.63, 7.00, 2.29).

Epigyne as in Fig. 16D–F; epigynal plate ~ 2× wider than long with two sclerotized and barely visible teeth; fovea small and almost rectangular; copulatory openings (*Co*) located on anterior edges of holes (Fig. 16D, E); copulatory ducts (*Cd*) with a membranous anterior part and a widened slightly sclerotized posterior part; receptacles tubular and twisted along their axis (Fig. 16F).

#### Distribution.

Known only from the type locality in Imereti Province, central-western Georgia.

#### Etymology.

The specific epithet refers to the historical Georgian polity of Egrisi, which was centered in present-day western Georgia.

### ﻿ Tegenaria hamid

Taxon classificationAnimaliaAraneaeAgelenidae

Brignoli, 1978

525EA0D6-22CE-5786-8E8D-F83E74DA4FC3

Figs 1D , 5A–D

Tegenaria hamid Brignoli, 1978b: 515, fig. 96 (♀).
Eratigena fuesslini : Topçu and Demircan 2018: 20, fig. 1 (♀).

#### Type material.

***Holotype*** • ♀ (MHNG), Turkiye: Isparta Prov.: Egridir, 18.04.1973 (P. Brignoli). [examined]

#### Other material.

Turkiye: Antalya Prov.: • 2 ♀ (ZMUT), Alanya, env. Kestel, Dim Valley, 36°32'34.5"N, 32°06'17.5"E, pine and oak forest, 2–9.01.2013 (Y.M. Marusik); • 1 ♀ (ZMUT), Asmaca, 36°36'32.3"N, 32°03'12.4"E, 686 m, pine and oak forest, 3.01.2013 (Y.M. Marusik).

#### Comment.

This species was previously known only from its original description. The single figure of the vulva provided by Brignoli (1978b) is rather schematic and not depicted from an exact dorsal view. Therefore, we present additional figures from various angles based on newly collected material from Antalya. Additionally, upon checking the record of *Eratigenafuesslini* (Pavesi, 1873) from Turkiye by Topçu and Demircan (2018), it became evident that the figure presented corresponds to the anteroventral view of the macerated epigyne of *T.hamid*, rather than *E.fuesslini*. Consequently, the record of *E.fuesslini* is hereby removed from the list of Turkish spiders.

#### Distribution.

Known only from Isparta and Antalya provinces, southwestern Turkiye.

### ﻿ Tegenaria hoeferi sp. nov.

Taxon classificationAnimaliaAraneaeAgelenidae

CECDEA3F-4947-509D-86A4-A7829ECA8B74

https://zoobank.org/3606E8DA-51F1-4AB9-81F1-6354AAA1456C

**Figure 25.**
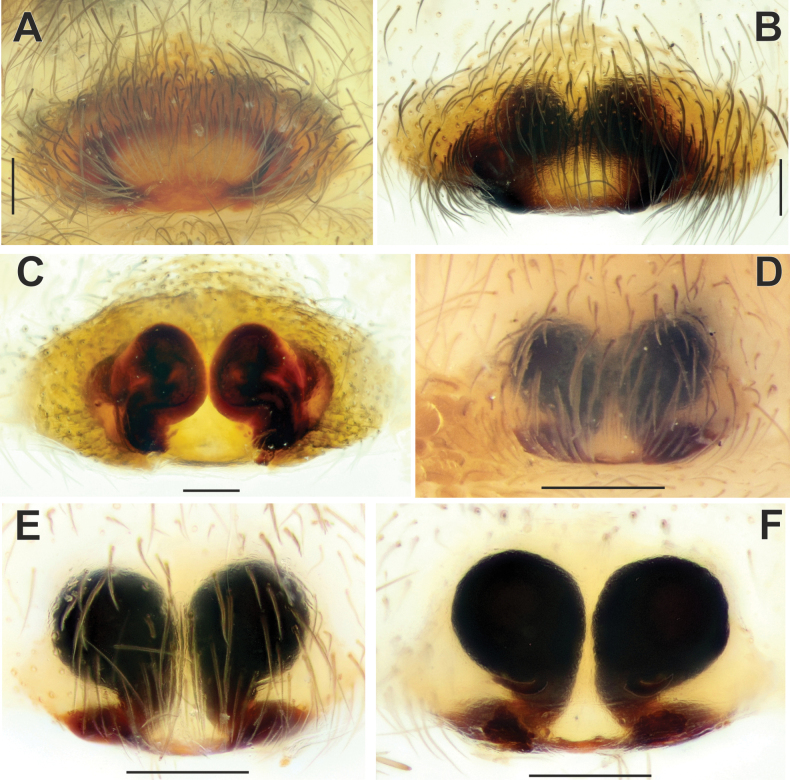
Epigyne of *Tegenariahoeferi* sp. nov. (**A–C**) and *T.tekke* (**D–F**). **A, D** intact, ventral view **B, E** macerated, ventral view **C, F** vulva, dorsal view. Scale bars: 0.2 mm.

Figs 23A–D , 24A–C , 25A–C

#### Type material.

***Holotype*** • ♂ (ZMUT), Armenia: Kotayk Prov.: env. Aghveran, 40°29'54"N, 44°35'24"E, 7–8.05.2021 (Y.M. Marusik). ***Paratypes***: • 1 ♂ 3 ♀ (ZMUT, ZMMU), same data as for the holotype.

#### Diagnosis.

The new species belongs to the *abchasica* species-group and is most similar to *T.chumachenkoi*. The male of the new species differs from that of *T.chumachenkoi* by the shape of the median apophysis, bulging proximally and widely pointed retrolaterally (vs straight proximally and sharply pointed retrolaterally in *T.chumachenkoi*; cf. Figs 23A, 15A). The female of the new species differs from that of *T.chumachenkoi* by having an oval median plate that is ~ 2× as wide as it is long (vs the median plate is not oval and is approximately as long as it is wide; cf. Fig. 25A and Ponomarev and Shmatko 2022: fig. 10).

#### Description.

**Male.** Habitus as in Fig. 24B. Total length 8.40. Carapace 4.10 long, 3.20 wide. Eye sizes: AME: 0.20, ALE: 0.21, PME: 0.17, PLE: 0.19. Carapace, chelicerae, labium, and maxillae pale brown; carapace with black submedian and marginal bands; sternum greyish brown, with yellow median lobulated band and six spots. Legs pale brown, with distinct annulations. Abdomen dark greyish, with numerous beige dots, patches, and stripes. Anterior spinnerets greyish basally and pale beige apically, posterior ones uniformly pale beige. Measurements of legs: I: 17.48 (4.56, 1.62, 4.06, 4.91, 2.33), II: 17.14 (4.40, 1.63, 4.07, 4.71, 2.33), III: 15.86 (4.29, 1.47, 3.57, 4.48, 2.05), IV: 19.60 (5.16, 1.52, 4.58, 6.08, 2.26).

Palp as in Figs 23A–D, 24C; femur roundly bent, ~ 4× longer than wide; patella swollen, approximately as wide as long with long dorsal seta almost as long as tibia; tibia ~ 1/2 as long as femur (not counting apophyses) (Fig. 24C); retrodorsal apophysis (*Rd*) approximately as long as tibia wide distally, with strong spine directed proximoventrally (Fig. 23B); retroventral apophysis (*Rv*) small (Fig. 23C, D); cymbium 1.8× longer than wide, tip ~ 1/3 of cymbial length, lacking distinct spine, with basal process (*Cp*); bulb as long as wide; median apophysis (*Ma*) large (Fig. 23A), bent prolaterally; conductor with long and thin distal arm not reaching prolateral 1/2 of cymbium; embolus with large base, free part originating at 9 o’clock position, thin, roundly bent.

**Female.** Habitus as in Fig. 24A. Total length 10.28. Carapace 4.00 long, 3.00 wide. Eye sizes: AME: 0.18, ALE: 0.20, PME: 0.16, PLE: 0.18. Coloration as in male. Measurements of legs: I: 16.95 (4.33, 1.69, 4.18, 4.55, 2.20), II: 15.92 (4.20, 1.63, 3.65, 4.15, 2.29), III: 14.43 (3.94, 1.43, 3.23, 4.00, 1.83), IV: 17.89 (4.74, 1.58, 4.21, 5.29, 2.07).

Epigyne as in Fig. 25A–C; plate 2× wider than long, median plate oval, wider than long (Fig. 25A, B); copulatory ducts and receptacles lacking distinct limits, contiguous (Fig. 25C).

#### Distribution.

Known only from the type locality in Kotayk Province, central Armenia.

#### Etymology.

This species is named after Hubert Höfer (Karlsruhe, Germany), a German arachnologist. He is the Curator of Invertebrates and head of Biosciences at the State Museum of Natural History Karlsruhe. He has made significant contributions to the study of spiders in both South America and Germany, leading numerous projects and helping to compile the largest dataset on distributions of spiders in Germany.

### ﻿ Tegenaria longimana

Taxon classificationAnimaliaAraneaeAgelenidae

Simon, 1898

1243443C-4259-5750-BE67-C741012C5D88

**Figure 19.**
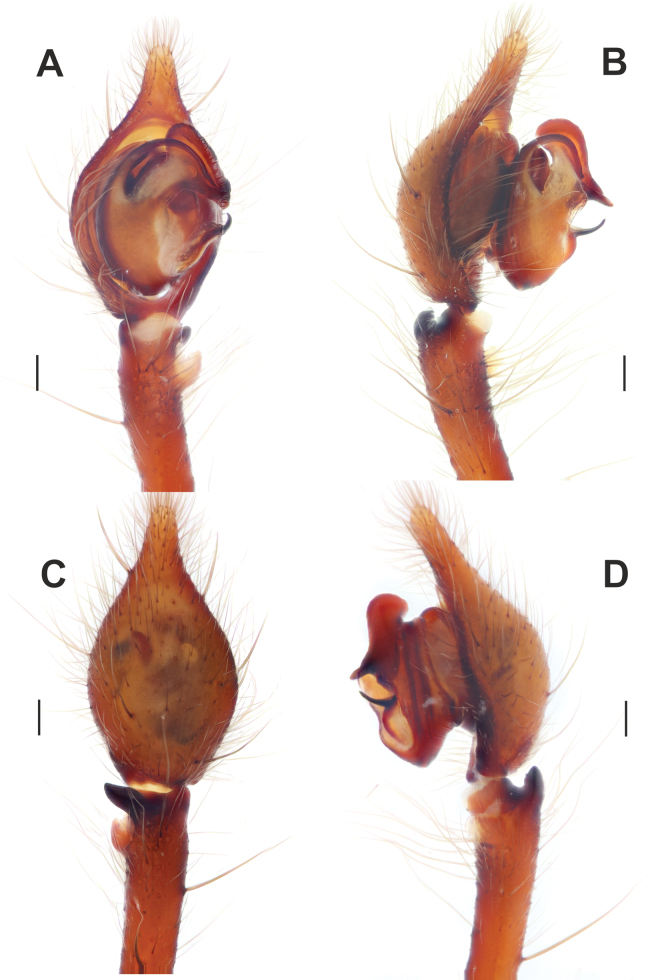
Male palp of *Tegenarialongimana*. **A** ventral view **B** prolateral view **C** dorsal view **D** retrolateral view. Scale bars: 0.2 mm.

**Figure 20.**
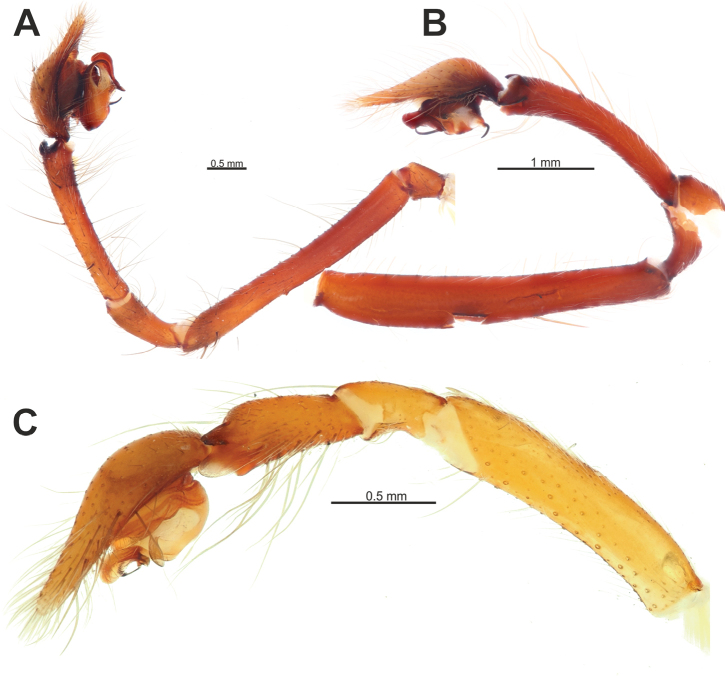
Full male palp of *Tegenarialongimana* (**A**), *T.anhela* (**B**), and *T.egrisiana* sp. nov. (**C**). **A** prolateral view **B, C** retrolateral view.

Figs 19A–D , 20A , 22C, D

Tegenaria longimana : Ponomarev et al. 2024: 279, figs 29–33 (♂♀).

#### Note.

For a full list of seven taxonomic entries, see WSC (2024).

#### Material.

Georgia: Imereti Prov.: • 1 ♂ 4 ♀ (ZMUT), env. of Tskhaltubo, Khomuli Vill., Tetra Cave, 42°19'49.3"N, 42°37'00.9"E, 454 m, 18.07.2012 (Y.M. Marusik); • 1 ♀ (ZMUT), Bzvani Vill., deep cave, near the entrance, 42°03'01.3"N, 42°36'04.5"E, 402 m, 20.07.2012 (Y.M. Marusik).

#### Comment.

This species was described from Batumi, the capital of the Georgian republic of Adjara.

#### Distribution.

Known from Turkiye, Georgia, and northern Caucasus (Otto 2022; WSC 2024).

### ﻿ Tegenaria percuriosa

Taxon classificationAnimaliaAraneaeAgelenidae

Brignoli, 1972

4B1447AE-7B4C-5B66-83FD-96B965D2913A

Figs 21A–D , 22A, B

Tegenaria percuriosa : Dimitrov et al. 2022: 3, figs 1, 2, 7–13, 40, 41 (♂♀).

#### Note.

For a full list of five taxonomic entries, see WSC (2024).

#### Material.

Turkiye: Antalya Prov.: • 1 ♀ 3j. (ZMUT), Alanya, env. Kestel, Dim Cave, 36°32'22.1"N, 32°06'34.4"E, 225 m, 4.01.2013 (Y.M. Marusik); Bursa Prov.: • 1 ♂ 1 ♀ (ZMUT), Uludağ, Göynükbelen rd., 39°59'N, 29°02'E, 14.05.2006 (R.S. Kaya); • 2 ♂ 2 ♀ (ZMUT), İnegöl, Great Oylat Cave, 39°56'N, 29°35'E, 519 m, 3.06.2009 (Y.M. Marusik); • 1 ♂ 1 ♀ (ZMUU), Uludağ Mountain, Baraklı Pond, 27.05.2003 (R.S. Kaya); • 1 ♂ (ZMUU), same, 8.05.2005 (R.S. Kaya); • 1 ♂ (ZMUU), same, 10.05.2010 (R.S. Kaya); • 1 ♂ (ZMUU), same, 1270 m, 16.05.2016 (R.S. Kaya); • 1 ♀ (ZMUU), Uludağ Mountain, Soğukpınar Valley, 40°03'N, 29°09'E, 5.06.2003 (R.S. Kaya); • 1 ♀ (ZMUU), Uludağ Mountain, Alaçam Forest, 30.08.2009 (R.S. Kaya); • 1 ♀ (ZMUU), Uludağ Mountain, Kocayayla Plateau, 25.09.2010 (R.S. Kaya); • 1 ♂ 14 ♀ (ZMUU), Uludağ Mountain, National Park, 40°06'N, 29°05'E, 2.06.2010 (R.S. Kaya); • 1 ♂ 2 ♀ (ZMUU), Kazanpınar Cave, 2.06.2009 (R.S. Kaya); • 2 ♂ 10 ♀ (ZMUU), same, 6.06.2009 (R.S. Kaya); • 2 ♂ 3 ♀ (ZMUU), Ayvaini Cave, 14.10.2012 (R.S. Kaya); • 1 ♀ (ZMUU), Oylat Cave, 15.10.2016 (R.S. Kaya); • 2 ♂ 2 ♀ (ZMUU), Mustafakemalpaşa Dist., Suuçtu Waterfall, 24.06.2012 (R.S. Kaya); Balıkesir Prov.: • 1 ♀ (ZMUU), Alaçam Mountain, 39°25'N, 38°35'E, 4.07.2012 (R.S. Kaya); Eskişehir Prov.: • 1 ♀ (ZMUU), Çatacık Forest, 39°57'N, 31°08'E, 1.08.2012 (R.S. Kaya); Isparta Prov.: • 1 ♀ (ZMUU), Zindan Cave, 21.05.2007 (R.S. Kaya); İstanbul Prov.: • 1 ♀ (ZMUU), Aydos Forest, 40°56'N, 29°14'E, 874 m, 30.04.2016 (R.S. Kaya).

**Figure 21.**
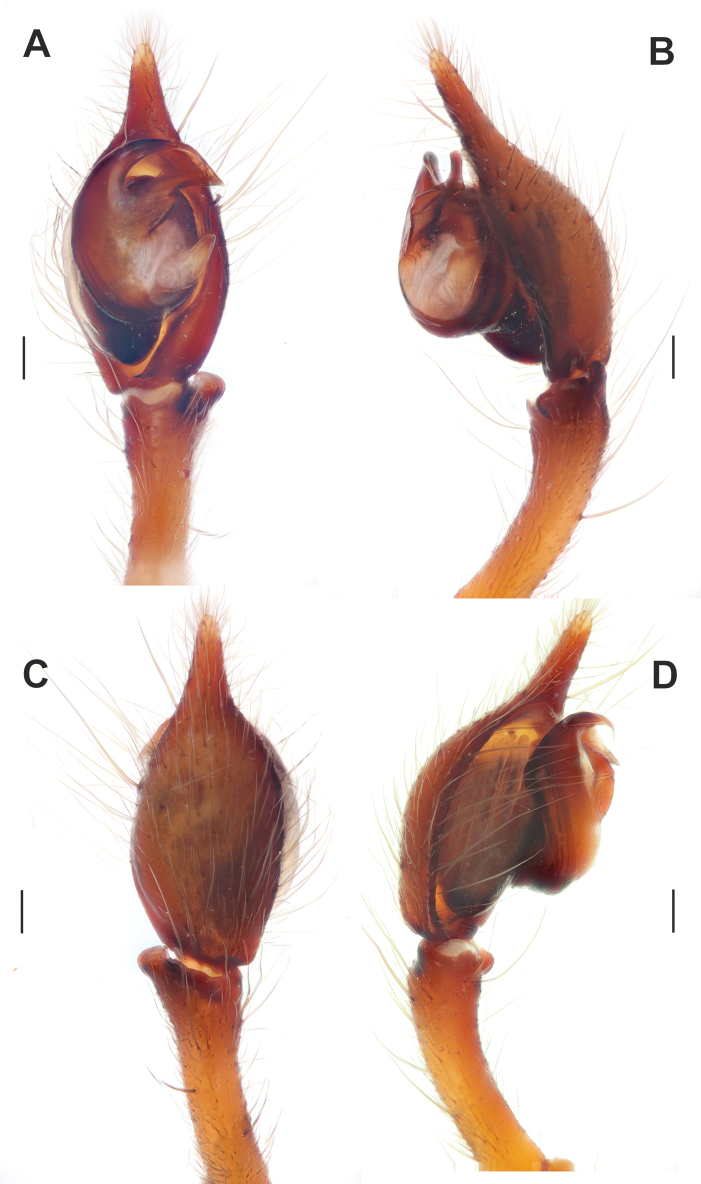
Male palp of *Tegenariapercuriosa*. **A** ventral view **B** prolateral view **C** dorsal view **D** retrolateral view. Scale bars: 0.2 mm.

**Figure 22.**
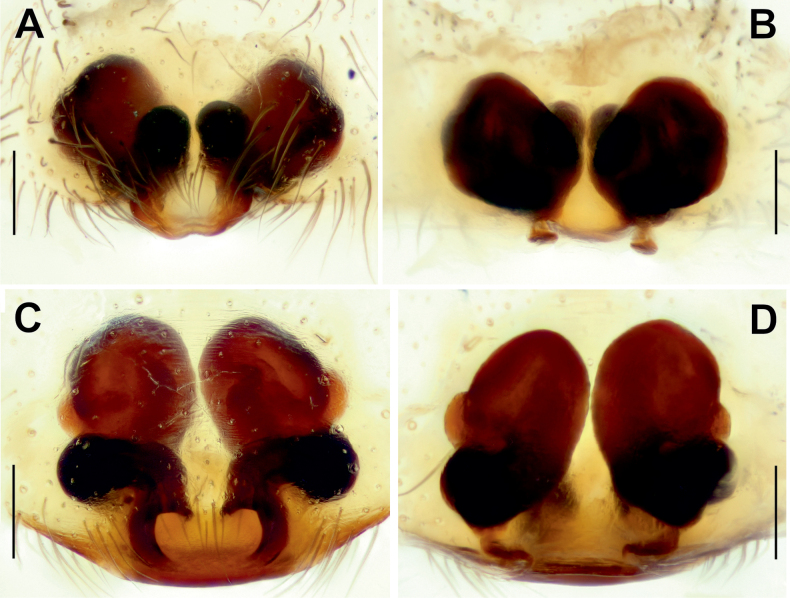
Epigyne of *Tegenariapercuriosa* (**A, B**) and *T.longimana* (**C, D**). **A, C** macerated, ventral view **B, D** vulva, dorsal view. Scale bars: 0.2 mm.

#### Comment.

In our male specimens, the tip of the apical part of the median apophysis is widened (Fig. 21A), which is different from that of the specimens illustrated by Dimitrov et al. (2022). This is herein considered an intraspecific variation.

#### Distribution.

Known from Western Anatolia (Dimitrov et al. 2022).

### ﻿ Tegenaria tekke

Taxon classificationAnimaliaAraneaeAgelenidae

Brignoli, 1978

2D2CA306-50F1-5890-A22E-E08519EB9FD7

Figs 18E , 25D–F

Tegenaria tekke Brignoli, 1978b: 516, fig. 98 (♀).

#### Material.

Turkiye: Antalya Prov.: • 3 ♀ (ZMUU), Kaş Dist., Kaş-Elmalı rd., 916 m, *Pinusbrutia* and *Quercus* sp. forest, 20.05.2012 (R.S. Kaya); • 2 ♀ (ZMUT), same.

#### Comment.

This species was previously known only from its original description.

#### Distribution.

Known only from Antalya Province, southwestern Turkiye.

## ﻿Discussion

As a result of this study, new taxonomic and faunistic data on the agelenid spiders of Turkiye, Georgia, and Armenia were provided. Turkiye is one of the most diverse countries in regards to Agelenidae, with 77 currently known species (including the results of the present study). This diversity is indeed higher compared to several other countries and regions, for example, the entire Caucasus (48 species), Greece (49 species), Bulgaria (44 species), Italy (58 species), France (41 species), and Spain (41 species) (Nentwig et al. 2024).

In this paper, four new species of *Tegenaria* were described, including two from Turkiye and one each from Georgia and Armenia. There are now 39 known species of this genus in Turkiye (Danışman et al. 2024; present study), and 32 from the Caucasus (Otto 2022; Ponomarev et al. 2024). The number of *Tegenaria* species recorded in each Caucasian subregion/country is as follows: Adygea (8), Armenia (1), Azerbaijan (15), Chechnya (3), Dagestan (7), Georgia (9 or 10), Ingushetia (1), Kabardino-Balkaria (0), Karachay-Cherkessia (3), Krasnodar Krai (13), North Ossetia-Alania (3), South Ossetia (3), and Stavropol Krai (6) (Otto 2022; Ponomarev et al. 2024).

Turkiye has been relatively well studied in terms of its Tegenariini, although new species and records continue to be discovered regularly. In the present study, all newly described species from Turkiye were collected from the Taurus Mountain range, a biodiversity hotspot located between the Mediterranean coastal region and the central Anatolian Plateau (Noroozi et al. 2019). The Taurus Mountains feature high altitudes, diverse valley slopes, depressions, and rugged karstic landforms, which create a variety of microhabitats and localized ecological conditions (Atalay et al. 2014). These mountain ranges in Anatolia, especially those divided by numerous valleys in the south, play a significant role in speciation and define various biogeographical subregions and provinces. From a zoological perspective, the Anatolian Taurus exhibits a very high degree of endemism and restricted local distributions (Çıplak 2003). This pattern of distribution underscores the importance of topography and microhabitat diversity in the evolution and distribution of *Tegenaria* in Anatolia. Many Anatolian species of *Tegenaria* are endemics, with most having limited, localized ranges primarily found in mountainous regions (Kaya et al. 2010). For instance, the fact that the two closely related species *T.bayrami* Kaya, Kunt, Marusik & Uğurtaş, 2010 and *T.ballarini* sp. nov. were both collected sympatrically from the same habitat further highlights the role of this region in speciation of this group. The Tegenariini of Georgia and Armenia remain less studied compared to Turkiye, although the region’s diverse habitats and topography suggest that many undocumented species are still waiting to be discovered.

## Supplementary Material

XML Treatment for Agelenini

XML Treatment for Persiscape caucasica

XML Treatment for Textricini

XML Treatment for Maimuna antalyensis

XML Treatment for Tegenariini

XML Treatment for Tegenaria anhela

XML Treatment for Tegenaria averni

XML Treatment for Tegenaria ballarini

XML Treatment for Tegenaria beyazcika

XML Treatment for Tegenaria chumachenkoi

XML Treatment for Tegenaria dalmatica

XML Treatment for Tegenaria egrisiana

XML Treatment for Tegenaria hamid

XML Treatment for Tegenaria hoeferi

XML Treatment for Tegenaria longimana

XML Treatment for Tegenaria percuriosa

XML Treatment for Tegenaria tekke

## ﻿Additional information

### Author ORCIDs

Alireza Zamani https://orcid.org/0000-0002-8084-9666

Rahşen S. Kaya https://orcid.org/0000-0002-3769-9105

Yuri M. Marusik https://orcid.org/0000-0002-4499-5148

### Data availability

All of the data that support the findings of this study are available in the main text.
